# Spirit of the Foreign Periodicals, &c

**Published:** 1841-01-01

**Authors:** 


### Spirit of the Foreign Periodicals.

### Interesting Case of Endo-pericarditis after Rheumatism.

On the last day of April, 1837, M. *Bouillaud* was summoned to visit Dr. R. residing 26 miles from Paris, who, he was informed, after having been for upwards of a fortnight suffering with severe articular rheumatism,* was then labouring under alarming symptoms of thoracic distress, so that his life was almost despaired of. On his arrival, he found his patient seated in an arm-chair, breathing with great difficulty, his face puffy and swollen, and his legs considerably infiltrated. Several medical men were in the room, and all of them were justly alarmed.

I examined, says our author, the respiratory and circulatory organs, and discovered the most characteristic symptoms of an endo-pericarditis (effusion into the pericardium) and of a double pneumonia with effusion into the pleuræ;—the effusion filled almost the entire left side, but only the lower third of the right side: the pulse was from 108 to 120. In spite of the advanced period of the disease, I at once recommended the adoption of vigorous antiphlogistic treatment.

The patient was bled immediately to the extent of sixteen ounces, and a large number of leeches applied to the chest, so as to obtain nearly twelve ounces

* In the letter requiring M. *Bouillaud's* presence, the following abridged account of the case up to that date is given. Dr. R. had on two former occasions suffered from acute gouty rheumatism. The present attack supervened about three weeks ago when he was not yet recovered from the influenza. Almost every joint in the body was affected with pain, and there was considerable fever, especially towards the evening; the pulse was not strong. The treatment consisted in the use of warm diaphoretics and gentle purgatives. Nine days ago, he was first seized with a sharp pain in the left sterno-clavicular region; it was relieved by leeches, but next day it returned with as great severity at the base of the chest on the left side, near the attachments of the diaphragm, and was accompanied with considerable embarrassment in breathing: it was considered a muscular pain. As however no relief was obtained from the application of leeches and afterwards of a blister, twenty ounces of blood were drawn from the arm, and the patient was put in a vapour bath. In spite of these means, the dyspnœa became more distressing; so much so that he was obliged to get out of bed and walk about the room: the pulsations of the heart were at this time extremely tumultuous. The dyspnœa was regarded as purely spasmodic; and, as the exacerbation of fever took place every evening, quinine was administered with the view of arresting the paroxysms. It is stated that, up to the evening of the 22nd April, auscultation afforded no symptom to indicate any alarm: that then for the first time a strong blowing sound was heard over the heart, the movements of which were excessively tumultuous. Next day, the patient was very low and depressed; his breathing was still very difficult, so that he could not lie down. He was cupped over the cardiac region; a blister also was applied there, and a mixture containing digitalis was ordered. The symptoms continued with increased severity up to the day that M. *Bouillaud* was summoned to his assistance. As each evening the dyspnœa became more and more alarming, and gradually abated towards morning, the physicians in attendance allowed themselves to believe that the disease was of an intermittent character, and required the use of quinine again, although it had been tried already without any benefit.

more. Next day, he was again bled from the arm—the blood on both occasions was buffy. During the night the patient was excessively restless and distressed, being every now and then forced to get out of bed in consequence of a feeling of imminent suffocation : occasionally too he was delirious. Towards morning he became easier, and his respiration more free, except when he attempted to move, and then it was always suffocating. The pulsations of the heart were violent but regular; the sound of œgophony was distinctly audible on the left side, but on the right it was no longer perceptible, and resonance was established at every point. The prognosis therefore was that the fluid in the right pleura had been absorbed, and the quantity in the left one diminished. Next morning (2nd May) another bleeding to the extent of eleven ounces was practised, and the blood was again found to be buffy ; the pulse was still upwards of a hundred, but the movements of the heart, though violent, were somewhat calmer. 3rd May. The patient was bled for the fourth time, and two blisters were applied on the chest —the œgophony was still distinct on the left side ; the evening exacerbation of the febrile symptoms was rather less. On the following day (4th) there was a marked improvement ; the pulse had fallen to 92, the hopes of the patient were raised, and he began to desire for food, having hitherto been restricted to " la diete la plus absolue." Every thing now advanced favourably; the level of the water on the left side gradually descended, and by the 15th, the fluid was nearly all absorbed. Two fresh blisters had been applied ; and in addition to the treatment already described the patient had, since the visit of M. *Bouillaud*, taken calomel in conjunction with digitalis or the extract of opium. Eventually Dr. R—— recovered his health completely ; and is now practising his profession at ———. *Traité du Rheumatisme par J. Bouillaud.*

The preceding history is very interesting, and illustrates exceedingly well the danger that is apt to arise from overlooking existent disease of the heart and from attributing to spasm the dyspnœa and other thoracic symptoms, when in truth they arise from inflammation. There is reason to believe that such an error in diagnosis was at first committed in the case of the late *King William IV.*, and that the disease had already advanced to a dangerous extent before antiphlogistic measures were resorted to.—*Rev.*

---

### M. ANDRAL ON ENDOCARDITIS AND CARDITIS—CASE OF ALLEGED GANGRENOUS ENDOCARDITIS.

.. ....... " The inflammation of the membrane, which lines the cavities of the heart, presents analogous appearances to those observed on the internal tunic of the blood-vessels when they are inflamed. The morbid changes are of a similar nature, although never existing to the same extent. Thus, the cavities of the heart are too large ever to be nearly obliterated from the effects of endocarditis ; but still there is no doubt but that a sudden and very manifest contraction of the cardiac orifices may be induced in this way. The internal surface of the heart exhibits a granulated aspect; and at the points which are the least smooth (les plus depolies) we observe small coagula adhering. If the endocarditis has become chronic and has existed for a length of time, the lining membrane becomes considerably thickened, and covered with large opaque patches, more especially in that portion which corresponds to the valves. These are the only morbid changes which are necessarily induced ; but others may be developed, such as cartilaginous concretions, or ossific cretaceous deposits. It is exceedingly rare that purulent matter is secreted on the surface of the inflamed endocardium.

In some cases the entire substance of the heart's parietes becomes affected with inflammation ; and, when this occurs, it is not unfrequent to find purulent matter under the endocardium, or fairly intermixed with the muscular fibres, which are then usually soft and lacerable. Sometimes we observe a genuinely ulcerative carditis; it may terminate in a complete perforation of the heart: when this occurs, the *ramollissement* of its substance is always very great. Such are the immediate consequences of carditis; among the more remote, hypertrophy of the organ is one of the most frequent. In truth, the orifices of the heart become contracted in carditis; to expel the blood, the organ must contract with more than usual force; and you know that it is a law of the organism, that every organ, whose activity is augmented, becomes the seat of a more active nutrition. Such is the usual cause of hypertrophic enlargement. In the same manner we find the walls of the stomach acquire a great thickening, when the pyloric orifice becomes in any way obstructed: the bladder also is liable to the same condition, when there is any impediment to the excretion of the urine."

............ The following case of what the observer, Dr. *Gigon* of Angouleme, calls gangrenous endocarditis, is reported in a recent number of the *Expérience.*

A man, 56 years of age, was attacked with symptoms of thoracic distress after exposure to wet and cold. He was bled twice from the arm, and various remedies were used. The dyspnœa, however, gradually increased, and at length Dr. G. was summoned to his assistance, during the sixth week of his illness; his case having been considered at one time as of asthma, and at another of hydrothorax, or of hepatisation of the lungs. When Dr. G. saw him, he was sitting upright, panting for breath, and every now and then crying out for fresh air, for he felt as if he was about to be strangled.

On examining the chest, the cardiac region was observed to be distinctly more prominent than the chest on the opposite side; the impulsion of the heart was scarcely perceptible; a distinct (beau) blowing and somewhat rasping noise was heard at both sounds; but it was loudest during the first sound towards the apex of the heart, and during the second one towards the edge of the sternum.

The auscultation of the breathing did not afford any thing remarkable; there was a slight mucous *rale* behind on the right side. The pulse was somewhat irregular, but did not exceed above 80 beats in the minute; it was soft, but not filiform. The diagnosis, which was formed, was *contraction of the right and left orifices of the heart, the probable result of overlooked acute endocarditis.* Dr. G. presumed that there was a contracted state of the cardiac orifices, from the distinct blowing and sawing noise heard during the sounds of the heart, and from the extreme difficulty of breathing that was present; and judged that this had been induced by preceding endocarditis, from all the details of the case. The attack was recent and acute—of about six weeks' duration at most. The patient had never suffered from rheumatism, nor experienced any distress about the region of the heart; and certainly a chronic organic lesion, capable of producing such alarming symptoms as were present, could not have allowed the patient to have undergone the fatiguing and laborious exertions to which he had been accustomed, before his attack of illness. He died three days after Dr. *Gigot's* visit.

*Dissection.*—In the right pleura there was a considerable effusion of reddish serosity; the lung on this side was pushed upwards and backwards towards the vertebral column. The left lung was adherent over a considerable extent; but its tissue, as well as that of the right one, was every where crepitant, although, when cut across, a quantity of water flowed out.

Immediately underneath their pleural surface there were numerous livid spots, very similar to those observed on the skin in purpura.

The pericardium was sound; and there was scarcely any fluid within its bag. The heart itself was large, and much distended with soft non-adherent coagula.

When freed from all its coagula, it weighed 13 ounces. The endocardium of the right cavities did not exhibit any appearance of redness or change any where; but the edges of the tricuspid valve were thickened and of a reddish colour at several points.

Around the orifice of the pulmonary artery the ventricle was coated with a tolerably firm layer of lymph; and this extended back among the calumnæ carneæ, and seemed to be continued from a coagulum, which existed in the auriculo-ventricular orifice. On the left side of the heart, the following morbid appearances were observed: the mitral valve was exceedingly strong and thickened: its edge was of a deep scarlet hue; but nothing indicated any imperfection of its mechanism. The chief lesion was in the aortic valves: two of them were indurated, and adhered together by their adjacent angles; they were thus immoveable, and projected considerably into the tube of the aorta. The endocardium at the bases of these valves was red and thickened; but towards their free edges, it was soft, friable, of an ashey-grey colour, and decidedly ulcerated. One of them was perforated at its centre by an opening, of a line in diameter. Every thing in the aspect of these two aortic valves indicated a gangrenous disorganization. There were no traces of ulceration on the third valve; it was only very red, and slightly thickened. These three valves exhibited, therefore, traces of distinct inflammation in three different stages;—1, redness with thickening; 2, gangrenous ulceration; and lastly, gangrene with perforation. The internal membrane of the aorta was reddened for a considerable extent beyond the valves; it was also soft, and could be easily separated with the nails.

Dr. *Gigot* appends the following remarks to the preceding details. "No doubt can exist, in our opinion, as to the date and the nature of the disease in the present case; for, six weeks before his death, the patient was quite well, and could engage in laborious occupation without any inconvenience. He had never suffered with any symptoms of pulmonary or of cardiac disease. All of a sudden, after exposure to cold and wet while perspiring freely, he was seized with sharp fever and difficulty of breathing: the treatment was inefficient, and he died.

Dissection revealed the following appearances: redness, swelling, softening, ulceration, gangrene, false membranes of the endocardium. With such symptoms as we have alluded to, and with these post-mortem appearances before us, we cannot refuse to admit the existence of acute and violent inflammation."

---

### M. ANDRAL ON THE CHANGES OF THE BLOOD IN DISEASE.

MM. *Andral* and *Ganneret* have for some years past directed their attention to the study of the blood, more especially in reference to the relative quantities of its fibrine, its colouring particles, and its serum, in a great variety of diseases; and the results of their enquiries were recently submitted to the Royal Academy. They are founded on the examination of 360 bleedings, drawn from 200 patients.

We may state, as one of the general results of their enquiries, that they have found the proportion of the fibrine, in 1,000 parts of blood, to vary from one to ten, that of the globules from 185 to 21, that of the solid matters of the serum or the albumen from 104 to 57, and that of the water from 915 to 725.

In a state of disease, it is not common to find that these different constituents of the blood increase or diminish in quantity simultaneously. Most frequently one only of them is decidedly affected; but occasionally two of them are altered in their relative proportions at the same time; and then this is usually in an inverse or opposite ratio. For example, when the quantity of the fibrine is increased, that of the globules is generally found to be diminished; and *vice versâ.*

Before stating the results of MM. *Andral* and *Ganneret*'s experiments, we shall briefly describe the composition of the blood in a healthy state, so that our readers may be enabled to compare the changes which occur in disease with the normal standard.

The *solid part,* the clot or coagulum, of the blood is composed of 1, the fibrine, which forms a large mesh-work holding in its interstices the other elements which enter into the composition of the clot; these are—2, the blood-globules—and 3, a great quantity of serosity, similar to that in which the clot floats, but which is retained in the meshes of the fibrine, as water is in the tissue of a sponge. The *fluid part,* or the serum, is water holding in solution —1, albumen—and 2, saline ingredients, the chief of which are the muriates of potash and soda, the carbonate of soda, the sulphate of potash, and the alkaline phosphates........ It is to be observed that, in the coagulation of the blood, the separation between its solid and its fluid constituents is far from being complete. The coagulum retains a large quantity of serosity,—amounting to at least three-fourths of its weight. It is therefore a most serious error to suppose that the proportion of the coagulum to the serum of the blood can be ascertained by a simple inspection of their respective quantities.......... To estimate the proportion of the fibrine, one of the best plans is to well beat or whisk the blood, while recently drawn, with a rod: if this be continued sufficiently long, all the fibrine will become attached to the rod, free from the red globules, and from the other constituents of the blood, which remain behind. ........ The globules are entirely distinct from the fibrine: the first may be altered, while the second remains unaffected; and vice versâ.

The globules are formed of albumen united to hæmatosine; there is another quantity of albumen held in solution by the serum. Whatever opinion we hold as to the nature of these two sorts of albumen, we cannot refuse to admit that one forms an integral part of the blood globules, and that it is quite distinct from the fibrine. The well-known experiment of *Müller* is decisive on this point. On filtering the blood of a frog, the globules are left on the filter, and we then procure a colourless fluid, from which there is gradually deposited a coagulum of pure fibrine. The old opinion therefore, that the globules of the blood consist of fibrine and colouring matter, is now universally acknowledged to be erroneous. Before we proceed to give a summary of the researches of MM. *Andral* and *Ganneret* on the various changes, in the relative quantities of the different leading constituents, of the blood in different diseases, we must have a standard of comparison to represent the condition of the blood in a state of health. The following may be assumed as sufficiently correct.

In 1000 parts,

| | | |
|---|---|---|
| There are 3 | of fibrine .......... | the dry Coagulum. |
| 2 | of hæmatosine ...... | |
| 125 | of solid albumen in the globules .......... | |
| 68 | of liquid or dissolved albumen | the Serum. |
| 12 | of saline matters .......... | |
| 790 | of water .................. | |

As it is in one or more of these elements that the manifold changes, which occur in the blood during different diseases, are manifested, it is most important to determine accurately the constitution of healthy blood. The errors which have been committed on this subject, even in recently published works, have been very egregious. For example, the proportion of fibrine has been stated to be as high as 10, 14, and even 24 in the 1,000 parts; and that of the serosity sometimes as low as 600!

But it is unnecessary to dwell further upon this subject; and we shall therefore now proceed to state, as summarily as we can, the results of MM. *Andral* and *Ganneret*'s observations on the changes which the different constituents of the blood undergo in different classes of diseases. These may be arranged as follow:

1. Those in which the quantity of the fibrine is constantly increased, as the *phlegmasiæ* and in tubercular *phthisis*.

2. Those in which the fibrine is in a normal or in a diminished quantity, while that of the globules is either normal or increased, as the *pyrexiæ*, and many *hæmorrhages* and *congestions*.

3. Those in which the quantity of the globules is always diminished, as *chlorosis*, &c. &c.

4. Those in which there is a diminution in the quantity of the albumen in the serum, as in *albuminuria* or the *morbus Brightii*.

It is not to be supposed that in many diseases the alteration of the blood is confined to one only of its constituents: several may be affected at the same time.

For example, if a chlorotic patient is seized with any inflammatory disease, the blood will be found still to contain a diminished quantity of the red globules, but then that of the fibrine will be now increased. "We have so repeatedly," say the authors, "witnessed these results, that, whenever the quantity of the fibrine is found to exceed five parts, we should not hesitate at once, and without further knowledge of the case, to assert that the patient is labouring under an inflammatory disease; and, on the contrary, whenever there is less than two parts of fibrine, we should, with equal confidence, deny the existence of such a complication."

Besides actual disease, the privation of nutritious food and any loss of blood will much influence the proportions of the constituents of this fluid: these causes act chiefly by diminishing the quantity of the red globules. But it is to be observed, that in different cases the influence of these agents is very different. There are in this respect great individual differences and a great inequality of resistance: so much so that while in one patient, from one bloodletting to another, the globules will not lose above two or three, in another patient the loss will amount to thirty or forty in a thousand parts. While the proportion of the red globules is always more or less affected by previous bloodletting, that of the fibrine is usually little, if at all, affected by it; it is very rarely diminished, and in some cases even seems to increase. It is only when the detraction of blood has been considerable, and when the proportion of the globules has commenced to undergo themselves a marked diminution, that we observe that the proportion of the fibrine, and indeed of the other solid constituents of the blood, becomes very sensibly impaired.

*First Class.*—MM. *Andral* and *Ganneret* have examined the blood in 82 cases of Phlegmasiæ—articular rheumatism, pneumonia, bronchitis, pleuritis, peritonitis, tonsillitis, erysipelas, cystitis, acute suppuration of the lymphatic glands, and furuncular eruption with fever—and in 152 bleedings practised in these diseases. In all these cases, where the disease existed in an acute form and was accompanied with fever, the blood drawn exhibited a notable increase in the proportion of its fibrine. If we take the number three as indicating the normal proportion in the thousand parts of this constituent in a state of health, in rheumatism, and in pneumonia, it was observed to oscillate between four and ten, the medium being seven and eight. In bronchitis the medium was between six and seven; in pleuritis and in peritonitis between five and six; and in the other diseases enumerated it was somewhat lower. In no case, however, did the proportion fall below four, and very rarely below five. It is to be remembered that in every instance the disease was strictly acute, and more over was attended with distinct febrile excitement. If the disease was originally, or if it had become of a chronic character, and if fever was never present, or if it had ceased, the proportion of the fibrine may no longer be found to be in excess.

Whenever a phlegmasia abates, the proportion of the fibrine in the blood diminishes; and if, after it has abated, it becomes again aggravated, the quantity of the fibrine is found again to increase. If an acute inflammation occurs in the course of another disease, its existence may immediately be detected by an increase in the proportion of the fibrine.

Very different is the case with the red globules; for it would seem that they never become increased in quantity in any inflammatory disease; often, indeed, they appear to be diminished. In all cases, however, when the inflammation subsides, the quantity of the globules is certainly diminished; but this effect is most probably owing to the loss of blood and the low diet which have been practised. A great diminution in the proportion of the globules does not seem to prevent the development of inflammatory action; and, on the other hand, a surcharge of them in the blood does not seem to predispose to or favour it.

The observations of MM. *Andral* and *Ganneret* shew that the development of inflammation is compatible with very different conditions of the blood in reference to the proportion of its red globules; this being sometimes as low as 48, and at other times as high as 60.

In all inflammatory diseases, the quantity of the solid constituents of the serum, or the albumen, did not exhibit any notable alteration. The proportion of the water varied from 771 to 840.

So much for the state of the blood in the phlegmasiæ. There is another disease in which our authors have invariably detected an unusual quantity of fibrine in the blood—viz. *tubercular phthisis. Whatever be the period of the disease, there is always observed a tendency to an increase of the fibrine and a diminution of the globules of the blood;* but neither the one nor the other change are at all equally marked in all its stages or phases.

As long as the tubercles are crude, the increase of the fibrine is inconsiderable, not exceeding four—the proportion in healthy blood being denoted by three; and the diminution in the proportion of the globules, although manifest, is not yet decided. But no sooner do the tubercles begin to soften, than the increase of the one constituent, and the diminution of the other, become much more apparent. At length when the lungs are hollowed with vomicæ, the proportion of the fibrine will be found to have risen to five, five and a half, or even six. If, however, the marasmus is very great, the quantity of this as well as of the other constituents obeys the law of decrease, and falls even below the normal standard. In general, the blood of phthisical patients contains the largest amount of fibrine, whenever they begin to be affected with a continued febrile movement.

While these changes are going on in the fibrine, the red globules are equally changed, but in quite an opposite manner: the proportion of these becoming less and less as the disease advances. During its first stage, the proportion of this element is at or above 100; in the second stage, it falls somewhat below this number; and in the third stage, it falls still lower, but it was never observed below 81. The solid constituents of the serum in phthisis were found to vary from 61 to 98; while the quantity of its watery portion varied from 784 to 845.

*Class Second.*—Diseases in which the fibrine of the blood is in a normal or diminished quantity, while that of the red globules is either normal or increased. This class embraces two orders—*pyrexiæ or fevers,* and several *congestions* and *hæmorrhages.* During the precursory stage of fevers, we never observe any increase, and rarely any diminution, in the fibrine; while, on the contrary, we never observe a diminution,—provided no blood has already been drawn—but often an increase in the proportion of the red globules.

*Simple Continued Fevers.*—Provided no distinct inflammatory action is going on in any part, the blood is usually found in the condition now described. In one case of well marked *angeiotenic* pyrexia, the proportion of the globules had risen as high as 185, while that of the fibrine was at the normal standard.

*Typhoid Fevers.*—In consequence of the inflammatory appearance of the intestinal lesions, which form the anatomical feature of these fevers, we might expect to find the blood exhibiting the characters which it presents in the phlegmasiæ; but such is not the case, whatever be the intensity of the intestinal inflammation. At no period of typhus is the blood ever found to contain a larger amount of fibrine than it does in health; but, on the contrary, the proportion is often decidedly less. The more serious the fever, the more decided is usually the diminution of this constituent. As to the proportion of the red globules, whereas this is but little affected at least before the employment of bloodletting, &c., it seems almost always in fevers somewhat higher than in health, more especially in the early stages of the disease. Thus even on the eighth day of a fever, it is not unfrequent to find the quantity of the globules as high as 140 or 150, whereas in pneumonia and acute rheumatism it seldom or never exceeds 130. Even after the employment of blood-letting and the use of a low diet in cases of fever, the proportion of the globules is seldom or never found so low as 130. How different from what we observe in the genuine phlegmasiæ! However,—and this circumstance is very remarkable—the high proportion of the globules either may never have existed or it may have ceased to exist, and yet typhoid fever may be developed and may proceed in its course.

*Eruptive Fevers,* as small-pox, scarlatina and measles. In these fevers, the fibrine of the blood has been found as low as one, and has never exceeded four; and this maximum was observed in one case only. It is indeed surprising that in a disease like small-pox, in which the skin is the seat of a profuse suppuration, the blood should never exhibit the signs which characterise this fluid in the genuine phlegmasiæ. We must therefore regard the cutaneous inflammation in variola, as well as the intestinal inflammation in typhus, as only one element of a more general disease which overpowers them, and from which the blood receives its peculiar characters. With respect to the globules in the eruptive fevers. We may state that we have found their proportion increased in several cases of rubeola and scailatina, but not in a single instance of variola.

*Intermittent Fevers.*—In all the cases in which the blood,—whether it was drawn during a paroxysm, or during an intermission—has been examined, our authors have obtained only negative results.

*Congestions and Cerebral Hæmorrhagies.*—In the majority of cases, but not in all, the proportion of the fibrine has been found below the normal standard, while that of the globules was either equal to or exceeded it: these characters were most distinct at the beginning of the invasion.

*Class Third.*—Diseases in which the quantity of the globules of the blood is invariably diminished. The most remarkable of these are chlorosis, various dropsies, the cachexia from the influence of lead, or from protracted agues, profuse hæmorrhages: in some cases of chlorosis the proportion of the red globules has fallen from 127 (the normal standard) as low as 38; more usually it does not descend lower than 50. In one case of extreme etiolation from profuse hæmorrhage, the proportion of the globules did not exceed 21. The proportion of the fibrine is never affected to the same degree as that of the globules in the diseases above enumerated. MM. *Andral* and *Gannaret* had repeated opportunities of observing the gradual increase in the quantity of the red globules in chlorotic patients, during the administration of steel medicines; in one case the rise was from 46 to 95.

*Class Fourth.*—Diseases in which the solid constituent, or the albumen, of the serum is diminished. MM. *Andral* and *Ganneret* have in several instances of albuminuria found that the proportion of the albumen in the serum of the blood had fallen from 72, the normal standard, down to 60 and 56. The other constituents of the blood have in these cases exhibited no steady or uniform deviations; those, which have been observed, seem to have been chiefly attributable to some accidental circumstance, such as the occurrence of inflammation somewhere, or of excessive weakness and emaciation.—*Gazette Medicale.*

It must be quite unnecessary to direct the *special* attention of every reader to the preceding most valuable communication. M. *Magendie* has already announced that he has been for some time past engaged in a similar series of experiments with those, which have now been communicated to the Academy by MM. *Andral* and *Ganneret.* The subject is one of exceeding interest, and of the greatest practical moment. We have of late years repeatedly expressed our conviction that it is chiefly by an accurate observation of the changes of the blood and of the secretions that we are to look for any important advancement in our knowledge of the nature of diseases, and of the most successful method of treating them. It might be hasty to draw any practical conclusions from the researches of which we have given a brief account: they require to be confirmed by other observers, before we are warranted in doing this. We may however direct our readers' particular attention to the very curious circumstance of the blood in phthisis exhibiting the same character, at least in reference to the increased quantity of its fibrine, as it is well known to do in the phlegmasiæ; and also to the very striking difference between the state of the blood in the phlegmasiæ and that of this fluid in the genuine pyrexiæ or fevers.—*Rev.*

---

### M. Rostan on the Pathology of Typhus Fever.

The following brief extracts from one of M. *Rostan's* recent clinical lectures will shew his opinion on the *gastro-enterite* doctrine of typhus.

. . . . . . . . " Is the affection of the Peyerian glands an inevitable occurrence in typhus, and is its existence essential to characterise the disease? We must admit that in an immense majority of cases, these glands are really affected in various degrees; but it is incontestable that the disease may exist without any appreciable post-mortem alteration in them. I should also mention that the Peyerian glands are not found to be altered in those diseases which have the closest analogy with typhus, such as phlebitis, and the absorption of purulent matter into the system. In the ataxic fever of Pinel all the characters of typhus are observed; but not its anatomical lesions. Far be it from us to call in question the existence of typhus; we only wish to shew that various causes may give rise to morbid poisonings, whose phenomena are similar to those of typhus, without their being attended with any alteration of the intestinal glands, such as is usually met with in it."

. . . . . . . . . . " The blood exhibits specific characters which are of the greatest interest. Those pathologists, who see nothing in typhus but a serious inflammation of the intestine, pretend that the blood retains at first all its normal properties, and undergoes alteration only in consequence and as a result of the disease, and then becomes diffluent. Now it is quite true that, at the commencement of the disease, the blood exhibits all its normal physical characters, and seems to retain all its physiological characters. In this respect it resembles the blood of persons who have been submitted to the action of the cow-pox virus. In them it apparently preserves all its properties; we cannot indeed detect any change or modification in it; and yet this cannot be strictly true, seeing that this blood has now acquired the property of resisting the influence of small-pox:—some change or modification therefore must surely have taken place in it. There is moreover something analogous in these two affections, cow-pox and typhus, and indeed in all other morbid states of the system which arise from the operation of any poisonous or septic agent. Thus the specific effects of the vaccine virus are not felt, during the first three or four days after its insertion, on the mass of the blood, which does not yet possess the properties necessary to resist the variolous contagion,—even although the vaccination may have already caused a febrile re-action. In the same manner in typhus, the blood retains, in the early days of the disease, its normal appearances, although most certainly it is already modified or altered by the influence of the typhoid poison introduced into the blood. These considerations lead us to believe that the alteration or modification of the blood, induced by the introduction of a poisonous agent, is gradual and progressive, and not simultaneous and immediate—the rapidity, with which this takes place, being probably owing to the greater or less virulence of the toxic or poisonous agent. I am of opinion, continues M. *Rostan*, that the fluidity or diffluence and the violet colour of the blood observed in typhus is not the result of the disease; but, on the contrary, that they are the immediate effect of the specific cause of the fever. Moreover experience demonstrates to us that the blood never undergoes these peculiar changes in any genuine inflammatory disease;—which should surely be the case if the modifications, which it exhibits in the course of typhus, were really the result of an open (*franche*) or prolonged inflammation, as many medical men of the present day believe."—*Gazette des Hôpitaux*.

*Remarks*.—We are glad to find that M. *Rostan*, whose name deservedly stands high among the physicians in the French metropolis, abjures the Broussaian dogma that typhus fever is in truth a *gastro-enterite*. We do not deny that the intestinal mucous glands are not unfrequently found, in this disease, more or less altered on dissection; but to regard such an alteration as the essential and initiative morbid element, is just as reasonable as to view the enlarged cervical glands the *cause* of the scrofulous diathesis. It is truly astonishing how long, and to what extent, so many of the French physicians have allowed themselves to be the dupes of a doctrine which *à priori* appears so improbable, and which, in spite of the zeal with which some of its advocates still support it, is gradually becoming more and more obsolete. We are pleased to observe that M. *Rostan*, while he exposes the fallacy of his countrymen in this respect, is impressed with the importance of studying the alterations of the blood in typhus fever. Every year we are returning more and more decidedly to the Boerhaavian hypothesis, that fever is the effect of some morbid poison introduced from without or generated within the system itself, and that its phenomena are just the evidences of numerous changes which the poisoned state of the blood induces in the numerous organs of the body. It is true that this doctrine in former times gave rise to many fanciful and most incorrect notions, which, as they were the offspring of mere conjecture and not of correct observation, can never gain our assent; but it is equally true that the humoral pathology of numerous diseases, and unquestionably of fevers among the number, gives a far more rational explanation of their phenomena than the modern doctrine of localisation, as the

French call it. That the exanthematous or eruptive fevers are the effect of the introduction of a poison into the system will not be disputed by any one. Then, why should not the same be the case in the case of typhoid fever, which has so close an affinity, nay, we may rather say, so perfect an identity, with them? The more that we consider the subject, the more reason we shall find to adopt a humoral pathogeny of typhus fever.

In a recent number of *Ammon's* Monatschrift für Medecin, &c., there is a paper by Dr. *Cramer* of Cassel, who maintains similar views. He says: "ordinary typhus is the result of an infection, or poisoning of the blood;" and in reference to the affection of the intestinal mucous glands, he says, "we must regard, as a peculiar product of the dyscrasis of the blood, the typhoid tubercle which is formed in these glands and excites ulceration in them. This ulceration is therefore one of the effects, and certainly not the cause, of the disease; it may be awanting, and yet the fever may nevertheless follow its usual course."

In another German journal, the Medicinisches Correspondenz-Blatt, for the same month, there is a paper on abdominal typhus by Dr. *Sicheser* of Heilbrunn. He considers it as "a disease of the blood," which attacks only secondarily the nervous and other systems of the body. In short, in every country, there seems to be a manifest tendency among medical writers to the establishment of a modern humoral pathology.—(*Rev.*)

---

### M. Rostan on the Treatment of Typhus.

M. *Rostan* very judiciously reprobates the adoption of any exclusive system of treatment in typhus. Alluding to the practice of *testing*, so to speak, the relative value of different modes of treatment by comparing their results in equal numbers of cases, he says,—

"Some physicians of late have taken, for example, a hundred patients, whom they have divided into five series, of 20 each, and then they have treated each series upon a particular plan, such as the antiphlogistic, the purgative, the tonic, or the expectant; and, from the comparative number of cures in each series, they have attempted to determine the relative value of these different modes of practice. Now what has been the result of this arithmetical mode of procedure? Why, that each mode of treatment has been nearly equally unsuccessful! But such a method of drawing conclusions is dangerous in the extreme; for each individual case of the disease requires an individual mode of treatment, and each form a peculiar management. Now each series having been taken at hazard, without any attention to the form of the disease, does it not follow as a necessary consequence, that a mode of treatment, which may have been suited for one form, was most probably any thing but appropriate to another form? It is thus evident that if those patients, who were affected with the inflammatory form, were treated with stimulants and tonics, they must almost necessarily have been injured; and on the other hand, if those, in whom the adynamic form was present, were treated antiphlogistically, an equally unsuccessful result must have followed."

M. *Rostan*, after these preliminary remarks, makes some brief comments on the different lines of practice which have been adopted in the treatment of typhus; and first on—

### *The Antiphlogistic or Depletory Method.*

Some physicians have pretended that by bleeding largely, and according to a particular formula, they have often succeeded in strangling the disease. From a considerable experience I am convinced that large and repeated bloodletting is the most certain method of inducing a most dangerous adynamic prostration and of accelerating the death of the patients. Where such a practice has succeeded, we may be certain that the case was not one of typhus fever, but rather of some genuine inflammation. I know, says M. *Rostan,* that to reason in this manner is to make a *petitio principii;* but my conviction is so strong upon this point that I am forced to admit such a principle. In all diseases which are produced by the action of some specific cause, bloodletting is insufficient to arrest the progress of the mischief; not so with respect to genuine inflammation. It is by reasoning in this manner that I can understand how those physicians, who do not recognize any *specificité* in typhus fever, and who regard it only as one form of gastro-intestinal inflammation, have succeeded in curing typhoid affections by bloodletting; for in truth the cases were examples of genuine inflammation, and not of fever. In fine, I am convinced that depletions of blood cannot be safely employed in the treatment of typhus, but with the greatest caution. In this respect I agree with MM. *Chomel* and *Louis* that, when the disease presents itself under an inflammatory form, we may have recourse to antiplogistic remedies with moderation, but that we should never exceed one or two small bleedings.

*The Expectant Method.*

M. *Rostan* mentions that, when the Academy of Medicine was recently occupied with the question as to the best mode of treating typhus fever, some of the Academicians submitted the results which they had obtained in their practice by "la methode expectante," or in other words by doing little or nothing, and thus leaving the case almost entirely to the curative efforts of Nature; and that it appeared that these gentlemen could boast of as many cures as their confreres who had been bleeding and purging their patients in the most vigorous style.

M. *Rostan,* however, is too sensible a man to allow his mind to be influenced by the reasonings of either party. The cures effected by the expectant method must have been in cases of but trifling severity, and where the febrile action had a tendency to run its course with moderation; but to suppose that either the strictly inflammatory, or the strictly adynamic form, of typhus is not under the influence of remedial measures, and should therefore be left to itself, is at once both most foolish and pernicious. It is, however, quite true that almost every case of fever, whether it be of a phlogistic or of an adynamic character, at one stage of its progress, requires an *expectant medication,* when all active treatment either of a depletory or of a tonic nature should be suspended. This is at the moment when the fever, having already received the application of the therapeutic agents which its type may require, has a natural tendency to progress in a favourable manner. Such a mode of *expectation* is, however, very different from the "fare niente" practice of some physicians. The practice in the one case is like that of a general who first strikes a decisive blow upon the foe, and leaves him to capitulate; that in the other, of one who expects that he will surrender without any effort made to compel him.

*The Revulsive Method, Cutaneous Irritants.*

M. *Rostan* at once proclaims his dissent from the opinion of M. *Louis,* that the use of vesicatories is hurtful;—he has repeatedly had occasion to observe that, after the action of a blister, the pulse has very considerably abated in frequency, and the other symptoms become diminished in severity. "I am convinced," adds our author, "that at a certain period of typhus, when the loss of blood cannot be safely continued, and when it would be unsafe to act on the system of *expectation,* we may have recourse to cutaneous revulsives with great propriety. It is true that they succeed less frequently than abstractions of blood; but we should remember that the proper period for their use coming after that for bleeding, and when this remedy has not sufficiently arrested the course of the disease that it may be safely left to itself, the conditions in which they (blisters) are employed are more unfavourable.

*The Purgative Method.*

.......... "Observing the little success that attended the practice of most physicians in typhus, M. *Larroque* came to the resolution of employing purgatives in all cases of the disease. Viewing the disease not as a simple inflammation, but as the result of a general poisoning or infection of the whole system, he imagined that the best plan of treatment must be to endeavour to destroy and evacuate the poison by promoting free evacuations from the bowels; and we must confess, that, by following out this idea, he has met with very considerable success in practice."

Is it not strange that an educated physician, like M. *Rostan*, should attribute to M. *Larroque* the introduction of the free employment of purgatives in typhus? The French writers seem to be utterly ignorant of what has been done in any other country save that of 'la belle France.'

The following sentence embodies the results of M. *Rostan's* experience of the purgative method. "In the first thirteen well-marked cases of typhus, in all of whom petechiæ had appeared on the surface, and in several of whom there were eschars on the sacrum, the employment of active purgation was attended with perfect success. Subsequently, however, we have not been so fortunate; and in truth, we have of late actually lost more patients by following M. *Larroque's* method than by any other plan of treatment." He proceeds to state that physicians differ much as to the most proper period of the fever at which purgatives should be employed; some recommending them at the beginning, and others at the close of the disease. (!) For his part, he adds, he has recourse to them, whenever there is the indication to purge, whatever may be the period of the disease at the time. (!)

*The Tonic Method.*

The employment of tonics for the cure of typhus has been most bitterly condemned by most physicians. In truth, they have been very unsuccessful; but then this arises from medical men seldom having recourse to them except in desperate cases; the tonic treatment is the *therapeutic extreme unction,* by means of which it has been expected to raise up patients from the profound adynamic state into which they have fallen. At the disastrous epoch of the retreat of our armies, the Salpetriere hospital was temporarily devoted to the military service. *Pinel* and *Landré-Beauvais* were at the head of the service, which I (M. *Rostan*) superintended for them at this period. During the space of six months, 18,000 patients affected with typhus were received into the wards; of this number, we lost only 1,100. All these patients were both physically and morally strong; most of them belonged to the old guards, picked men, who for many years had been accustomed to victory, but they had been exposed to privations, and to defeats besides; and they were now beaten by men whom they had always seen fly before them, and their *morale* was profoundly affected. Who would have dreamed of treating such men with bleeding and other depletory measures? We put almost every one of them on a generous treatment, and administered wine, camphor, musk, &c.

You will perhaps object that these cases were examples, not of genuine typhoid affection, but of nosocomial typhus, and what is suited to the one is not the proper treatment for the other disease. Do not deceive yourselves upon this point; it is all the same disease; the essence of both is the same; they only differ in degree."—*Gazette des Hôpitaux.*

---

### PATHOLOGY OF INTERMITTENT FEVERS.

In a work published last year in Paris, and which has been very favourably noticed in some of the French journals, "Traité des Fievres ou Irritations Cerebro-spinales intermittentes," by Dr. Maillot, lately one of the physicians of the French army in Algiers and now professor of medicine at Metz, the doctrine that the primary seat of the morbid action in agues is in the nervous centres is maintained with great ability, both by reasoning on the symptoms of the disease, and by the more sure test of necroscopical examination. Several dissections are reported, and the lesions of the cerebro-spinal axis are described with great accuracy. Other writers, before M. *Maillot,* have adopted somewhat similar views; and some have gone so far as to define an intermittent fever to be a periodic cerebro-spinal irritation; but to him is due the merit of having illustrated this position by post-mortem researches.

That an intimate connexion exists between intermittent fevers and various forms of neuralgia is sufficiently obvious from the genuinely periodic character of numerous cases of the latter disease. It is scarcely necessary to add, that the same remedy, bark, which is a specific for the one, affords as infallible relief in the other. The analogy therefore between ague and neuralgia affords much curious matter for speculation; and when we call to mind that so many of the worst cases of the latter disease are unquestionably connected with some affection of the nervous centres, at a distance therefore from the seat of the actual pain, we are to a certain degree led to suspect that a similar lesion may possibly give rise to that singular series of phenomena which constitute a paroxysm of intermittent fever.*

---

### ON THE ORIGIN OF CANCER IN THE VEINS.

Dr. *Langenbeck,* of Gottingen, informs us that he has been led by numerous microscopical examinations of diseased tissues to the belief that the seat of cancer exists very frequently in the venous system. He is however not ready to assent to M. *Cruveilhier's* opinion, that cancer is in every case developed primarily and essentially in the veins. In two cases of cancer of the uterus, followed by cancerous formations in the lungs, he found within the pulmonary veins carcinomatous matter—recognizable with the microscope by its peculiar cellular form—either free, or adhering to the parietes of the vessels. The molecules of the cancerous matter, carried along in the venous stream, may be arrested at any point, and there become developed, and acquire an increase, just in the same manner as any other organic molecule which, under a cellular form, is augmented in volume—as proved by the observations of *Müller, Schwann,* and other physiologists on cellular productions (êtres) of the vegetable as well as of the animal kingdom.

The cancerous elements may find their way into the circulating fluid in three different ways;—either they may be engendered in the blood itself, and, being carried along with it, are at length stopped at some point where they become developed; or a cancer may be formed in some of the solid tissues, and a portion of the fluid with which it is impregnated becomes absorbed by its veins or lym-

---

* We may refer the reader for some excellent remarks on the analogy between intermittent fever and neuralgia, to Dr. *Billing's* admirable First Lines —a work in which there is more original thought than in any other we have met with for the last twenty years.—*Rev.*

phatics, and is thus conveyed into the circulation, giving rise ultimately to the formation of new cancerous growths ; or, lastly, cancers already ulcerated may corrode the veins or lymphatics of the part, and thus the cancerous cellules (which are each of them germs of new cancers,) may be introduced into the current of the circulation.

The author admits that he has not been able to demonstrate experimentally in any case the primary formation of cancerous cellules in the blood; but, judging from numerous microscopical examinations of the blood in persons already affected with cancer, he thinks that such development is highly probable.

As to the second and third modes of explanation, it is to be remembered that the internal surface of veins, which arise from any organ affected with cancer, is very generally found corrugated, and more or less morbidly altered ; thus the disease is apt to be developed in the veins of the liver, if the primary evil has its seat in the extremities of the vena portæ ; and in the veins of the lungs, when it is *en rapport* with the two venæ cavæ.

In two cases of cancer of the uterus, the author has seen, within the veins of the pelvis, the cancerous matter of *Cruveilhier* under the form of minute grains or cellules of coagulated fibre of about double the size of pus-globules. In the blood in the iliac veins also, as well as in the inferior cava and the right cavities of the heart, there were observed here and there, by means of the microscope, cellules inclosing nuclei of a yellowish colour, and numerous granules like those which were found in the pelvic veins ; in the capillaries of the lungs too the carcinomatous granules were very abundant and were more adherent to the walls of the vessels than elsewhere ; and in the pulmonary parenchyma itself new cancerous foci were discovered. In reference to these facts, the author makes the curious remark, that " the cancerous germs or cellules transported to another organ are there developed into a voluminous cancer, just as the germ or ovule of the ovarium, when conveyed into the uterus, becomes developed into the fœtus." In short, he looks upon the cellules of cancer as veritable organised beings, which only require to be fixed in a suitable place to develop and multiply themselves ; and he therefore so far agrees with those writers who have designated cancers " parasitical tissues."—*Schmidt's Jahrbucher.*

---

### On the Seat of Gonorrhœa in Women.

M. *Gibert,* in a memoir recently read before the Academy, thus expresses his opinion.

. . . . . . " In women, as in men, the seat of election of blenorrhagia is the meatus urinarius ; but in all the cases, where we have been able to use the speculum, we have observed, at the same time, a uterine discharge co-existing with the urethral flux, and usually remaining for a length of time after this (the latter) has ceased ; so that, in my opinion, we must regard the cervix uteri as the principal source of the blenorrhagia in women.

Several modern writers, deceived by a superficial observation, have most improperly designated the disease by the term of vaginitis. Now in a large majority of cases of gonorrhœa, the vagina remains unaffected (*etranger*), and if it occasionally is found to be reddened and inflamed, this is only temporary, and speedily subsides by rest and cleanliness. It is only in rare and exceptional cases that we observe a milky or puriform discharge proceeding from the mucous membrane of the vagina. On the contrary, in every woman, who has contracted gonorrhœa, there exists during the first two or three weeks a characteristic flow of pus from the urethra ; and subsequently a discharge comes from the cavity of the cervix uteri, and this usually lasts for a length of time after the urethral discharge has ceased, and ultimately cannot be distinguished from ordinary leucorrhœa or fluor albus." ........

"Out of 216 cases of gonorrhœal flux in the wards of our hospital of Loureine, a great many of which were of several weeks' standing at the time of their admission, we have been able most distinctly to make out the presence of urethritis in 88, and the presence of any affection of the vagina in 40 cases only; and, no doubt, the number of the former would have been much larger if the examination had taken place at an earlier period of the disease; while in reference to the latter number it is to be observed that in many of the cases the redness of the vaginal mucous membranes very quickly disappeared."

---

### M. BEAU'S NEW THEORY OF THE SOUNDS OF RESPIRATION.

"The object of the present communication is to support with new facts the theory which I proposed in 1834, (Archives Generales) as to the cause of the respiratory sounds, and to show the various applications of which it is susceptible, by passing in review all the normal and abnormal sounds produced in the larynx, the bronchi, and the lungs. This theory rested on the following two propositions.

1. There is produced in the superior respiratory passages a sound or murmur which resounds in the pulmonary vesicles, the trachea, the bronchi, the caverns, and which, in consequence of this resonance, is the only cause of the different sounds, known under the names of the *vesicular*, the *tracheal*, the *bronchial*, the *cavernous*.

2. Every sound produced in the superior respiratory passages must resound in the bronchial tree with its proper character and its own degree of intensity.

These two propositions were deduced from clinical observations and from several experiments, of which the following are the chief:—

1. When the superior sound is suspended,—and this may easily be done by instinctively dilating the superior respiratory passages,—the vesicular, tracheal, bronchial and cavernous sounds can no longer be heard. The breathing, although now very tranquil and silent, goes on as usual; but if we did not feel under the ear the thoracic parietes alternately rise and fall, we might suppose that the person did not breathe.

2. If we suspend the superior sound during one only of the respiratory movements, then the vesicular, tracheal, bronchial and cavernous sounds are found to be suspended during that movement, while it is still audible during the other.

3. If we produce a whistling sound either during respiration or expiration, the same kind of sound is heard in the bronchial tree.

Dr. *Spittal* has in part adopted these views, and has published the results of several experiments which he has made. He concludes:—"It is therefore reasonable to admit that the sound of the superior respiratory passages exerts, and must exert, a certain influence on the sounds of respiration, known under the names of vesicular, bronchial, cavernous, amphoric. But the preceding experiment is not of a nature to demonstrate that the superior sound is the only source of the different sounds which have been enumerated, although we are strongly inclined to think so."*—*Edin. Med. & Surg. Journal, Jan.* 1839.

---

* The first idea of this theory belongs to M. *Chomel*, who, in 1827, used the following words in treating of the blowing sound which accompanies pleuritic effusions:—"*Laennec* is of opinion that the sound of bronchial respiration is owing to the inspired air being stopped in the bronchi, which are compressed and flattened by the effusion into the pleura. But then how should this sound

It has been objected by MM. *Raciborski* and *Stokes* of Dublin, that the superior respiratory sound is often suspended, and yet the vesicular murmur is still audible. It is quite true that such *seems* to be sometimes the case; but in truth it is not, and the mistake arises from want of due precision in making the experiment. The superior sound, although scarcely perceptible, is not completely suspended; and the remnant of it, so to speak, however feeble, is still made resonant in the pulmonary vesicles. To avoid all chances of deception, it is necessary to perform the experiment in a very quiet room; and moreover another person should auscult the larynx, to ascertain whether the superior sound continues or not, while the experimenter is listening to the pulmonary sounds. By attending to these rules, we shall find that no vesicular murmur can be detected without a superior sound being present, and that these two sounds are always proportionate in distinctness. We are therefore entitled to conclude that the vesicular murmur is the result not of the friction of the air in the pulmonary cells, as supposed by *Laennec* and most writers, but of the resonance of the superior sound transmitted along to them. This view is confirmed by the following experiment: adapt a pig's bladder, moistened, to the end of a tube whose diameter is the same as that of the trachea,and then put the other end of the tube into the mouth, and after closing the nostrils breathe only into the tube and bladder: the bladder is observed to be alternately distended and relaxed. The person can, at will and alternately, suspend or exaggerate the superior sound. If the thorax be ausculted at this time, we find that the pulmonary sounds are alternately null or exaggerated, and yet, in these successively different conditions, the bladder does not exhibit any difference in the extent or rapidity of its movements of distention and relaxation. The inference therefore is fair that the air is sent as quickly, and in as large a quantity, into the pulmonary vesicles when the vesicular resonance is audible, as when it is not.

The question to be now considered is, where is the origin or seat of the superior respiratory sound, which, when re-echoed in different parts of the bronchial tree, gives rise to all the sounds emitted by the organs of breathing, alike in health and disease? The orifices or contractions of the respiratory passages capable of causing vibrations in the air which traverses them, and of producing the superior sound, are five—the lips, the nostrils, the isthmus of the pharynx, the glottis, and the upper opening of the larynx.

1. *The Lips.*—If we open the lips so that the interval between them represents an area of 14 or 15 centimetres, there is produced a sensible sound on inspiration and expiration. The sound increases, if the interval is straightened; and it is no longer audible, when it exceeds 15 centimetres.

2. *The Nostrils.*—They are susceptible of only a slight contractility. When they are dilated as much as possible, and the lips are closed at the same time, there is scarcely any sound heard during breathing. If, on the other hand, they are contracted, so that the total area of the two orifices is below 14 centimetres, we perceive a sound, the intensity of which is proportionate to the degree of the contraction. With respect to the posterior openings of the nostrils, as they are immoveable, and moreover considerably larger than the exterior openings, the air must traverse them without occasioning any sound.

3. *Isthmus of the Pharynx.*—This orifice, of a considerable extent, may be

---

be heard during expiration? Is it not more probable that it is produced in the larynx and the back of the mouth, and that it is transmitted to the ear in the same manner as the voice, which is produced and articulated in the same organs?"—*Dict. de Medecine.*

contracted by lowering the velum palati, and by elevating the tongue. It requires, however, a certain practice to contract it so that the passage of the air through it gives rise to a sound.

4. *Orifice of the Glottis.*—This opening is essentially moveable; its area, when the inferior vocal chords are separated from each other, is about 15 centimetres. This area may be progressively diminished until it becomes completely closed; and it may be so much dilated that it equals that of the larynx. When the area of the glottis is normal, a distinct sound is produced by the air traversing it; its intensity increasing or diminishing, according as this is contracted or dilated.

5. *Opening of the Larynx.*—The area of this orifice is about 40 centimetres. If the lips, the pharynx, and the glottis, be dilated as much as possible, and the respiratory rhythm be normal (16 inspirations in the minute), then no sound will be produced in the upper respiratory passages. But if we quicken the breathing beyond 45 inspirations in the minute, there is produced a sound, whose greatest intensity is audible at the upper part of the neck, and which can be produced only at the opening of the larynx. The lips, pharynx, and glottis being dilated as much as they can be, the opening of the larynx, which is immoveable, becomes the most contracted point of the respiratory passages: and this orifice which, in consequence of the extent of its surface, gives out no sound when the air traverses it slowly, produces a distinct one whenever there is any disproportion between the volume of the air admitted and the area of the laryngeal aperture—such as takes place whenever the breathing is much quickened.

From what we have now said, it would seem that as it is the *glottis* alone, which continually presents an obstacle to the transmission of the air, causing it to enter into vibrations, it must be to it that we must refer the origin or seat of the upper respiratory sound. Let us remark also that the seat of this sound, being the nearest possible to the lungs, is thereby well fitted to produce a resonance of it along the bronchial tree; whereas other sounds, and chiefly the nasal and labial ones which are the outermost of all, reach the interior of the chest with difficulty. It is this *glottic* sound, therefore, reverberated in the air-tubes and pulmonary vesicles, that gives rise to all the various sounds of respiration described by auscultators. It is a double sound, at one moment *inspiratory,* at another *expiratory.* To study these properly, we must not be satisfied with listening at a distance; we should auscult the larynx itself. The relative duration of the two *glottic* sounds is determined by the duration of the corresponding respiratory movements of the chest.

And therefore, as the act of inspiration occupies in health nearly double the time of that of expiration, it follows that the *duration* of the inspiratory glottic sound is about twice as long as that of the expiratory sound. Again, the *intensity* of the former is notably less than that of the latter: a circumstance which confirms the truth of the statement by *Le Gallois,* that the glottis is more contracted during expiration than during inspiration. (These remarks, be it remembered, apply chiefly to the adult.)

The two *glottic* sounds are audible at a considerable distance. By means of auscultation, we may perceive them on the back of the neck, and even on the top of the head; but it is in the trachea and in the lungs that their resonance presents the most interesting phenomena. This resonance is nearly as great in the trachea as it is in the larynx; but it becomes less and less distinct as we recede from the glottis and approach the lungs; and in these it not only diminishes from the upper to the lower part of the chest, but its character is very sensibly altered. It is no longer a blowing sound, but rather a diffused murmur, caused by the vibration of the air in the innumerable minute divisions of the bronchi, and in the pulmonary cellules.

M. *Beau*, after alluding to the well-known circumstance that the resonance of the expiratory sound in the lungs is not only shorter, but also much less distinct and of a less marked vesicular character than that of the inspiratory sound—although the former is louder in the larynx than the latter—explains the cause of this difference in the following sentence:—

"This very marked difference of intensity between the resonance of the inspiratory and that of the expiratory glottic sounds, cannot be well explained by M. *Laennec's* theory, according to which the vesicular murmurs are supposed to be directly produced by the friction of the air along the bronchial mucous membrane. For, it may be asked, why should the air, on leaving the pulmonary vesicles, not cause as much friction, as on entering them? In truth, the friction must surely be greater, seeing that the act of expiration is performed more quickly than that of inspiration; and if so, then the vesicular murmur ought to be stronger—which however is contrary to fact."

*The Voice.*—The voice is the distinct sound produced by the expired air passing through the contracted glottis, and reverberating outwards. But while its chief resonance is outwards, it also penetrates, at the same time, backwards, and resounds along the whole extent of the bronchial tree, in a direction contrary to the current of the air.

It is this inward resonance of the voice that at present we have to consider. When we auscult the voice over the larynx and trachea, we hear each syllable distinct and well articulated, but over the chest the resonance much less clear; there, it is rather a confused and prolonged murmur. The reason of this difference is owing to the trachea being superficial, with nothing to intervene, whereas the bronchi are deep-seated and surrounded more or less completely with the soft parenchymatous tissue of the lungs; and hence, as the air is quitting the vesicles at the moment that the vocal resonance takes place, it must follow that the sound thus produced will be more or less confused and deadened.

This is so true that, when the voice is produced during inspiration—which may be easily done after a little practice—the vesicular resonance of the voice is found to be much more complete and more distinct than it is during expiration.

From these observations it must be apparent that there is a much greater analogy between the voice and the ordinary sounds of respiration than has been usually supposed: both are produced in the glottis, and both result from the vibration of the air passing through the vocal chords.

They are both also, to a certain extent, under the influence of the will, and are therefore susceptible of increase and diminution; but the voice is by much the loudest in its resonance, because not only is the aperture of the glottis more contracted, but the volume of the air is expelled with more force during utterance, than during ordinary respiration.

We shall now follow out this analogy, by tracing the effect of different diseases on those two kinds of *glottic* sounds.

With respect to the voice, it is well known that, whenever the pulmonary parenchyma becomes impermeable to the air, either from hepatisation, or from tubercles, or from pleuritic effusion, &c. the vocal resonance becomes louder and tubular—this is called *bronchophony*.

A still louder and more distinct resonance is heard when the voice is reverberated in a cavity—this is *pectoriloquy*. These sounds are only two different degrees of a circumscribed and tubular resonance of the voice, and they are chiefly dependent on the diameter of the normal or the abnormal cavities which produce them. That genuine pectoriloquy will sometimes proceed from a simple bronchus, especially if it be dilated and surrounded with hepatised tissue, when there is no excavation in the lungs, is confirmed by daily observation. On the other hand, a cavity may exist in the lungs, and yet there may be no distinct pectoriloquy, but only a bronchophonic resonance.

With respect to the other abnormal resonance of the voice, *œgophony*, we must confess that it is not easy to explain it. This sound is most frequently perceived when there is an effusion into the cavity of the pleura. Occasionally, however, it is heard when the trachea or one of the large bronchi is compressed by a tumor, such as an aneurism of the aorta, &c.

The œgophonic resonance of the voice is also heard in some cases of bronchocele.

We proceed now to consider—

*The Glottic Sounds in an Abnormal Condition.*—They may be increased, diminished, or perverted.

*Increased Sounds.*—Whatever causes an unusual disproportion between the aperture of the glottis and the volume of the respired air, has the effect of increasing the force of the glottic sound.

This disproportion exists—1, when the air traverses the glottis very rapidly—and 2, when the glottis is more than usually contracted. The *first* of these two conditions accounts for the greater intensity of this sound in children, and in some women, also after exercise, and in diseases which affect the breathing, such as pneumonia, pleurisy, consumption, &c.; and the *second* explains the occurrence of the same phenomenon in cases of spasm of the glottis, which is so frequent in hysterical patients.

We have already seen that, when the breathing is quickened beyond forty inspirations in the minute, there is produced an abnormal sound at the superior orifice of the larynx. This sound is added, in this circumstance, to the ordinary glottic sound; but the former is the weaker of the two, in consequence of the upper opening of the larynx being less contracted than that of the glottis itself. In certain cases, however, when the laryngeal opening is much contracted, as in œdema of the aryteno-epiglottideal ligaments, it becomes very loud. Other pathological states may induce an abnormal contraction in certain points of the larynx or trachea, and give rise to sounds, whose character is similar to that of the glottic sound.

In this way, sub-mucous abscesses, or swelling of the laryngeal cartilages, or the presence of tumors in the neck, &c. may be accompanied with such sounds in particular parts of the windpipe. These different sounds, altogether abnormal in point of situation, may have the usual character of the normal glottic sound; or they may be associated with other sounds, which then more or less completely disguise it.

*Diminished Sounds.*—The glottic sounds are diminished in intensity, or even almost extinguished, in circumstances the very opposite to those mentioned in the preceding sentence, viz.—1, when the respiration is very slow, and the air passes through the glottis very gently, as during syncope; and, 2, in certain varieties of hysteric dyspnœa, where the glottis is unnaturally dilated.

*Perverted Sounds.*—It often happens that the sounds, produced either at the glottis, or at some abnormally contracted part or accidental opening of the larynx or trachea, lose their usual blowing character, and acquire a certain snoring or whistling tone:—we shall allude to these varieties by and bye.

If we examine the relative *intensity* and *duration* of the two—the inspiratory and the expiratory—glottic sounds in disease, we shall find that the latter one continues, as it is in health, always stronger than the former. There is scarcely an exception to this remark, except in cases of œdema of the glottis, where the inspiratory is always much louder than the expiratory sound. As to the relative

*duration* of the two, we meet with the following differences :—1. The inspiratory sound is, as in health, longer than the expiratory one ; but it may be so in a very abnormal ratio, such as we observe in certain spasmodic states of the glottis, and in œdema of the aryteno-epiglottideal folds ; 2, in other cases, the expiratory is much longer than the inspiratory one, as in common asthma : and 3, the two sounds are sometimes of equal duration, as in all cases where the respiration is quickened, in pneumonia, the fits of ague, &c.

" From what has now been stated," says M. *Beau,* " it follows that, whenever the glottic sound is very feeble, or altogether extinguished, its resonance must in like manner be the same. We are not, like *Laennec,* to conclude from this, that in such circumstances the air is received very feebly, or not at all, into the lungs, but only that it reaches them without producing any sound at the glottis. When, on the contrary, the glottic sounds are exaggerated, the resonance will be so likewise—the result of the passage of the air through the glottis being more difficult, and therefore more noisy, than in health."

He objects to the common explanation given of the occurrence of the *puerile* respiratory sound during pneumonia in those parts of the lungs not affected with the inflammation. " To admit," says he, " that a larger quantity of air than usual enters the sound parts of the lungs, we should be prepared to shew that in such circumstances the respiratory movements, the extent of which must be regarded as the strict measure of the quantity of inspired air, are of unusual amplitude. Now, in truth, we do not observe anything extraordinary in these movements on the healthy side of the chest ; and on the inflamed side they are almost suspended, paralysed, so to speak, by the acute pleuro-pneumonic pain. And yet, even on the affected side, where the respiratory movements are so obscure, there are frequently considerable portions of the lung which are not inflamed, and which nevertheless give out a puerile murmur, the intensity of which is quite as great as that heard on the healthy side."

M. *Beau* accounts for the occurrence of the puerile respiration in pneumonia by the greater intensity of the *glottic* sounds, in consequence of the more rapid passage of the air through the glottis. The breathing becomes more frequent, to compensate for the loss of function in the inflamed parts of the lungs : it is only in this sense that we can justly say that the healthy parts make up for the deficiency occasioned by the impermeability of the inflamed portion. The same cause—the frequency of the respiration—which produces the puerile murmur in young children, gives rise to it not only in pneumonic patients, but also after exercise, in phthisis, during the paroxysm of agues, in many fevers, in spasm of the glottis, and in various diseases and in accidental openings of the larynx and trachea, &c. In all these cases, it is dependent upon the greater force of the resonance in the pulmonary vesicles of the exaggerated laryngeal or glottic sounds.

This is the proper place to allude to an important mistake which we find in the great work of *Laennec.* It is there stated that, " whatever be the efforts of inspiration made, a healthy adult cannot give his respiration that degree of intensity which it had in infancy." Now so far is this from being the case, that we do not hesitate to assert the very contrary, and that in all inspirations, short or long, a healthy adult may give to his pulmonary sounds a puerile intensity. All that is necessary for this purpose is only to increase the force of his inspiratory glottic sound—which it is most easy to do. It will perhaps be objected to this statement that *Laennec* has most distinctly denied this, for in one part he says :—

.......... " At other times patients, imagining that we are asking of them something extraordinary, endeavour to dilate the chest with all their power, or rather they make several deep inspirations, without expiring in the interval, and in such cases we scarcely hear any sound at all." This remark of *Laennec* is quite true ; some patients, when told to breathe deeply, make several strong, almost convulsive, inspirations. But if we attend minutely, we shall find that these exaggerated inspirations are accompanied with a dilatation of the glottis, which has the effect of at once silencing the *glottic* sound, and therefore its resonance or reverberation in the vesicles of the lungs is no longer audible. *Laennec* himself seems to have felt the difficulty of explaining the fact in question; for he admits, a few lines subsequently, "that the pulmonary sounds imply the existence of an action proper to the lung itself, and which is not necessarily connected with a deep inspiration."........The theory of *Laennec* on the production of the respiratory murmur, which leaves, as we have seen, several facts quite unexplained, is not more fortunate in accounting for the *puerile* sounds. To reconcile them with this theory, we have been called upon to admit the gratuitous hypothesis, that there is a greater penetration of the inspired air into the vesicles of a healthy, than of an inflamed, portion of lung. But how shall we account for the well known circumstance of the vesicular sounds being so much exaggerated in certain cases of contraction of the larynx or trachea, and also in cases of accidental opening in these tubes? The explanation of *Laennec* here quite fails. On the contrary the *theory of resonance*, which we have proposed, indicates very satisfactorily the mechanism of all exaggerated pulmonary sounds, by showing that they are the result of whatever increases the force of the laryngeal or upper respiratory sound. It is therefore with no little surprize that I have observed, says M. *Beau*, that Dr. *Stokes* of Dublin, in his recent work on the diseases of the chest, objects to my theory on the ground that it does not give any explanation of the *puerile* sounds. I cannot understand the ground on which this author makes his objection.—*Archives Generales.*

*Remark.*—As the memoir of M. *Beau* seems to us one of the most valuable that we have met with for a long time in the French periodicals, (the contents of which are usually so unimportant) we have been induced to give a very extended abstract of it. A continuation of it is promised by the ingenious author. —(*Rev.*)

---

DISPARAGEMENT OF AUSCULTATION, BY M. LUGOL. ORIGIN OF TUBERCLES.

M. *Lugol*, during the course of last summer, delivered a series of lectures on scrofulous diseases. The following is an extract from the fourth lecture on the formation of tubercles in internal organs :—

............ "The numerous checks, and repeated deceptions to which physicians are daily exposed in the diagnosis and treatment of tuberculous diseases, do they not prove that it is necessary to leave the beaten track of enquiry and pursue some other which is less fallible? You all know that *auscultation and percussion are useless in the diagnosis of pulmonary tubercles.* Both alike insufficient to announce the commencement of the mischief, they are superfluous at the very time that they become capable of indicating the presence of the tubercles; for then these are discoverable by other means, and alas! are too far advanced in their development to warrant our hopes of arresting their progress —at least in the generality of cases. I will even go a step farther, and say that the unlimited confidence, placed by the greater number of practitioners of the present day in auscultation and percussion, has had the effect of too often inspiring a fatal security in many tuberculous diseases, which are thereby allowed to advance in their progress, until this is revealed by physical phenomena at a period when remedial measures have but little chance of effecting any good.

"But what are the means, you will say to me, that are to be substituted in the room of auscultation and percussion? I answer, gentlemen, induction. Examine by these boasted methods this patient, and tell me what results you obtain. Negative results, you will reply. And yet I maintain that he is tuberculous; for his father, his mother, and his brothers have all died of tuberculous disease; and he himself is affected with it in his chest at the present moment. Believe me, this plan is much less deceptive than the other one. I tell you, the inductive method cannot mislead you; for nature is invariable in its causes as in its effects; and the external signs of tuberculous scrofula must give you assurance that similar morbid productions exist in internal organs, and especially in the lungs.

It is by viewing the question from this elevated point of view, by studying it in all its *ensemble,* that you will be best enabled to comprehend it in its details; and these cannot be understood by the special methods of examination which have been so much recommended of late years.

The tuberculisation of internal organs exhibits in its development the same phenomena as tubercles which are outwardly situated—there is no pain, and nothing of mechanical derangements."

In a subsequent part of the same lecture, M. *Lugol* thus treats of *Tubercles of the lungs.* "The existence of tubercles in the lungs is so frequent, that I must admit that they are present in all scrofulous persons. You know that all, or almost all, patients, who have pulmonary tubercles, are, or have been at some time, affected with tubercles in the neck; the majority have had during infancy this external sign of scrofula; while others have had it at a later period of life. I believe that pulmonary tubercles frequently exist in early youth; but it is chiefly about the age of puberty that they are apt to be developed. Puberty in truth seems to have a fatal specific influence in promoting their development; and in our wards at the present moment there is a case which seems to confirm this opinion. A scrofulous patient, who, although 22 years of age, exhibited none of the usual characters of marriageableness; he has just died; and in him no tubercles were found in the lungs.

Sometimes however pulmonary tuberculisation seems to disappear about the period of puberty, and, ceasing for a number of years, it does not again develop itself perhaps until the 40th or 50th year of life."

M. *Lugol* subsequently alludes to the probable origin or *genesis* of tubercular formations. He regards them as *parasitical organs,* the development of which takes place *par intus-susception.* (Query. Should not this be rather intus-*conception*?) "They are not," says he, "mere degenerated organs, as many authors suppose; for if they were so, we should be able to distinguish the tissues of these organs in the tubercles, while they are still incipient. Far from this being the case, we observe that a tubercle is always the same from the very commencement of its formation, and that the organs, in which it is developed, impress no modification upon it, except perhaps upon its size and form."

After stating his opinion that inflammation does not engender, but merely promotes, the development of tubercles, he adds:

"It is impossible to see any other thing in tuberculisation than a sort of parasitical function, which establishes itself by the side of some normal function, and to its detriment. This *epigenesis* is not more difficult to understand than that of hydatids, intestinal worms, lice, &c. with which it has a great affinity, and which are often found re-united in tuberculous children. A great analogy, I repeat, exists between the creation of lice, intestinal worms, and tubercles. All three are subject to *peripeties,* which seem to have some connexion with certain individual and atmospheric conditions. I have frequently seen spontaneous and abundant generations of lice take place in persons, who have subsequently died of tuberculous disease."—*Gazette des Hôpitaux.*

### Orfila's recent Experiments on Poisoning.

The following summary of the distinguished toxicologist's researches, which have been detailed at greater length in the two last Numbers of the Medico-Chirurgical Review, is taken from a recent number of the *Moniteur.*

M. *Orfila* commenced his experiments to-day, in one of the amphitheatres of the Faculty of Medicine, before the members of a Committtee of the Academy and a numerous audience. The Professor made a number of experiments on poisoning with arsenic and with tartrate of antimony, and, by the programme which he distributed, he undertook to prove—

1. That these poisons, when introduced into the alimentary canal, or inserted into the subcutaneous cellular substance, are absorbed, and mingle with the blood, and are thus carried through all the organs of the body.

2. That they remain for a certain time in the substance of the different viscera and of the muscles, where their existence can be demonstrated by chemical processes; but that, from the time of the poisoning, a portion of that which has been absorbed leaves these tissues, and is eliminated by the urinary secretion.

3. That this elimination, which is much more rapid in the case of the antimonial than in that of the arsenical salt, continues during several days, until the tissues in question have been completely freed from the presence of the poison.

4. That it is therefore advantageous, and even indispensable, in the treatment of poisoning from these salts to promote the urinary secretion.

5. That it is possible in most cases to distinguish whether the arsenious acid and the antimonial tartrate, which are withdrawn from the viscera, have been absorbed during life, or have entered the body through *cadaveric imbibition* after death.

6. That the most convenient process for detaching minute quantities of these absorbed poisons consists in destroying the major part, or the entire, of the organic matter of the viscera and muscles, by carbonising them with pure concentrated azotic or nitric acid, and introducing the products into *Marsh's* apparatus somewhat modified.

7. That it is always easy to distinguish arsenic from antimony by the form of the spots left on the vessel, and at the same time to ascertain that these spots do not proceed either from the apparatus itself, or from the chemical tests employed.

8. That there exists in the bones, both of man and of certain animals, an arsenical compound insoluble in water.

9. That from the muscular flesh of the human body may be extracted a matter, which M. *Orfila* believes to be formed of exceedingly small proportions of arsenic, sulphur, and an organic substance.

10. That in the earth of some cemeteries there are found quantities, exceedingly small indeed but appreciable, of arsenic, which boiling water will not dissolve.

11. Finally, that it is easy, in a legal enquiry, to avoid those errors which might appear, at first sight, likely to arise from the admitted fact of the presence of arsenic in human bones and muscles, and in the earth of certain cemeteries.

M. *Orfila* then proceeded to make his experiments. Several dogs were poisoned; in some the salts were introduced into the stomach, and in others they were inserted under the skin: the latter method proved most rapidly fatal. The following results were obtained.

1. The urine of the dogs which had been poisoned yielded, when submitted to *Marsh's* apparatus, distinct traces of the metallic salts; while that of other dogs, to which no poison had been given, did not exhibit any traces of them.

2. A small portion of the liver of the poisoned animals—having been previously charred with nitric acid, and the residue introduced into the apparatus—yielded numerous spots of arsenic ; while the entire liver, spleen, and heart of a dog killed by hanging, on being submitted to the same chemical treatment, did not exhibit any traces of the metal.

---

### On Gastromalacia, or Softening of the Stomach in Infants.

It is scarcely ten years ago since the medical journals began to report for the first time any cases of this disease ; although Dr. *Jaeger* of Stuttgard drew the attention of physicians to it in 1811. (Vide *Hufeland's* Journal for 1811 and 1813).

In the Summer of 1831 it shewed itself, with all the characters of an epidemic, at Gottingen and the adjacent districts ; and its symptoms were then so well marked that, since this period, it has been impossible to mistake it for any other disease, even during the life of the patients.

Dr. *Winter* has published an excellent work on Gastromalacia, for which he received in 1833 the prize of the Royal Scientific Society of Gottingen ; it was published in the succeeding year, and has contributed much to our more accurate knowledge of its pathology. The author of the preceding and following remarks, Dr. *Iselin* of Mullheim, has met with twelve cases of the disease, and, having also perused all the descriptions given of it by different writers, he is now induced to submit them to the notice of his professional brethren.

*Description of the Disease.*—According to my observations, says he, infants under twelve months of age are most subject to softening of the stomach ; but it is certain that it is occasionally met with at any period of youth up to puberty.

*Cammerer* indeed (Versuche uber natur der Krankhaften Magenerweichungen) says that he has seen it even in adults. However this may be, it is admitted by all pathologists, that it is much more frequent during the first year than at any other period of life.......... With respect to the duration of the disease, this is found to vary very considerably : sometimes it proves fatal within twenty-four hours, while in other cases it has lasted for several weeks......... According to its different characters, the disease is either *idiopathic* or *symptomatic.* Idiopathic Gastromalacia is either *acute* or *subacute.* In the first, the infant is seized with high febrile heat, vomiting and purging of serous acid matters ; the features shrink, the eyes are sunken, and life is extinguished within twenty-four hours from the attack. In the *subacute* form, the disease commences usually with purging and generally also with vomiting ; the febrile re-action of the system may be inconsiderable ; and hence the symptoms may continue for several weeks.

Symptomatic Gastromalacia is usually preceded either by hydrocephalus, or by an acute exanthematous disease, or by some disease of the respiratory organs. In these cases, the course of the disease is almost always very rapid.

*Symptomatology.*—The disease exhibits two distinct degrees or stages—that of irritation, and that of palsy.

*First Stage.*—After two or three days of restlessness and general distress, a smart fever sets in, accompanied with great thirst, loss of sleep, and violent vomiting which cannot be checked. These symptoms are followed by a diarrhœa, at first of grey-coloured thickish matter, and then of a yellowish serosity which is found to be very acid. The face becomes sometimes very pale, at other times it is red and flushed, the features are often spasmodically contracted, whenever the bowels are purged, or when the belly is pressed upon; there is usually complete anorexia, and a greater or less degree of tympanitic fulness. The whole abdomen, and more especially the ventricular region, is felt to be very hot, while at the same time the extremities are usually cold; the infant is continually crying and moaning; and often we observe partial sweating on the surface, especially on the back of the head, which is in general very warm.

*The Second Stage* is marked by frequent accessions of sudden prostration, amounting almost to a state of syncope; the breathing is short and much distressed; the pulse is very frequent and irregular; the child no longer cries, but keeps moaning in a sort of stifled manner; the pallor of the face increases, and the eyes become sunken; the sweats are more general; the belly remains warm while the extremities are cold; slight convulsions come on; sometimes there is squinting, and at other times the eyes are fixed, and only half covered with the lids. The purging and vomiting often subside or cease altogether, and the appetite returns for some time before death. When the child drinks, a peculiar noise is heard in the belly; aphthæ form in the mouth; the face acquires a bluish hue, especially around the mouth and eyes. Most frequently there is no coma, nor any loss of consciousness; but towards the close an excessive prostration, accompanied with frequent syncope and a very rapid pulse, so that death may occur without being perceived.

The *Subacute* form is usually attended with a diarrhœa, which resists all attempts to check it; sometimes vomiting is present and sometimes it is not. The course of the disease is more slow, and the child ultimately sinks, perhaps after more than once seeming to be about to recover.

The same thing happens when softening of the stomach occurs as a secondary and symptomatic disease,—in this case the symptoms become well marked only when the organ is very seriously altered.

On the whole, the tumefaction of the abdomen, and the great heat of its surface, while the extremities remain cold, are perhaps the most constant and characteristic symptoms of the disease. The former continues after death, so that the position and form of the stomach are often visible through the abdominal parietes. In addition to these symptoms, the peculiar expression of distress in the features, and the sunken state of the eyes, most striking indeed in the second stage of the disease, are rarely absent in any case.

The acute form is always attended with fever; in the chronic form, there are frequently intermissions; the groanings, the restlessness, the convulsive twitchings of the face, and the oppression, are almost constant symptoms in both.

The diarrhœa and the vomiting are, on the contrary, not so. In some cases there is constipation. The evacuations are always acid, although they vary much in consistence.

Convulsions characterise the stage of palsy; loss of consciousness is rare; aphthæ are frequent.

*Anatomical Characters.*—In three cases examined by Dr. *Iselin,* the following appearances were found.

The abdominal cavity was distended with gas, and the cellular tissue of its parietes was emphysematous; the stomach was projecting and tympanitic, and the intestines were in the same condition. In two of the cases, the stomach was perforated in three different places; in the other case, it was not perforated, but its tissue was so soft that it yielded on the slightest effort. In the cul-de-sac, especially where it is contiguous to the spleen, were observed transparent and colourless patches, on which the muscular fibres, almost in a diffluent state, were visible. The rest of the stomach was not much altered, except in the neighbourhood of the cardiac orifice.

Around the softened patches, there were distinct traces of inflammation.

The softening takes place from within outwards, and the disease seems to begin with inflammatory action; the softening and the dissolution of the mucous membrane seem to be the result of it: the muscular and the serous tunics are subsequently affected. The intestinal canal, in my examinations, exhibited several inflamed and softened spots in different places. In one case, the duodenum was completely diffluent, as well as several isolated patches in the ileum. The mesentery was always inflamed and its glands in a state of engorgement. In every one of the three cases, there were several intus-susceptions of the bowels.

The morbid appearances in the other viscera vary in different cases, none of them being constant or characteristic.

*Causes of the Disease.*—The *predisposing* causes seem to be—1, a cachectic state of system induced by bad nourishment, and the generation of acid in the stomach; 2, a hereditary cachexia, the result of a phthisical or other morbid condition of the mother during pregnancy; 3, dentition; 4, any sudden change in the food of the child, or weaning suddenly and at an improper time; and 5, the passions of the mother, which, by altering the properties of the milk, are so apt to derange the health of the child.

*Jaeger* knew a woman, who was of an impetuous character, and often suckled her children during a fit of passion, lose three of them from softening of the stomach.

With respect to the influence of poor food in inducing this disease, it is not easy to determine this influence exactly; for some children, to whom every attention is paid, die of it; while many, who are ill and irregularly fed, escape.

Perhaps the most influential exciting causes are certain, unknown indeed, atmospheric changes; since the disease has been generally observed to prevail at the same time as bilious fever, dysentery, ague, &c.

With respect to the more immediate causes of softening and perforation of the stomach, *John Hunter* attributed it, in almost all cases, to the direct influence of the gastric juice itself, operating as a solvent on its walls after death; but this opinion, although no doubt quite true in cases of sudden death occurring during a state of health, is certainly not admissible in such cases as we have been alluding to:—the softening is the result rather of a general alteration of the organism during life.

*Cammerer* has shewn, by experiments on animals, that vinegar softens the coats of the stomach; and also that the contents of a softened stomach, when introduced into the stomach of another animal, have the effect of softening this last—provided the influence of the nervous system has been previously paralysed either by death, or by division of the nerves.

*Jaeger* also, (*Hufeland's* Journal for 1811 and 1813,) alludes to a chemical action of the gastric juices; but he does not believe that this action can operate as long as the stomach retains its normal condition, and supposes that some other agency must always be co-existent. The gastric juice is, according to his opinion, altered by certain morbid states of the nervous system, and in this way there is generated an acid, which has the power of softening the stomach.

*Heischmann* and *Meckel* have adopted nearly the same views—with this difference, that the former of these writers regards the spleen as the source of the formation of the acid. *Lenhossek* is of opinion, that the symptoms of the disease, at least in its earliest stage, are those of a cerebral affection—which acts sympathetically on the stomach, so that this organ loses its power of resisting the solvent action of its own juices; at the same time it is probable, says he, that the excess of acid, produced by the cerebral affection, acts sympathetically on the juices of the alimentary canal.

*Chaussier* (Bulletin des Sciences Med. No. 53,) and *Laisné* (Medecine Legale,) consider the disease as an erosion or ulceration of the mucous membrane of the stomach, that is incessantly increased by the contact of the ichor secreted on the surface of the ulcer.

*Rudolphi*, in his Grundriss der Physiologie, suggests that the stomach of infants, when it is affected with disease, is subject to a sort of putrefaction, and that the commencement of the softening is preceded by an acid fermentation.

*Winter* regards softening of the stomach as a disease of the general organization; and this opinion is confirmed by the existence of a number of different maladies which are often associated with it—such as the exanthemata, erysipelas, induration of the cellular tissue, jaundice, aphthæ, inflammation of the abdominal viscera, phthisis, hydrocephalus, &c.

The simultaneous existence of gastric softening, and of the diseases now mentioned, makes *Winter* believe that the cellular tissue should be regarded as the propagator of the morbid process; inasmuch as it contains the capillary system in which the disease begins. As to the morbid matter, which is at once the principle and the agent of this alteration, we must seek for it in the blood itself.

Dr. *Iselin* is of opinion that the primary cause of the disease is inflammation; but he does not adduce any facts to establish his views.

*Treatment.*—What we know on this subject is most unsatisfactory.

*Jaeger* admits that he has failed in every case, in spite of blisters, sinapisms, friction with stimulating substances, the internal use of carbonic acid gas, opium, musk, zinc, &c. &c.

Dr. *Iselin* says that much may be done in the early stage of the disease, that of irritation and fever, by leeches to the stomach and head, and the use of antiphlogistics. Mercury and alkaline medicines* are always hurtful, he adds, while oleaginous demulcents are very useful.

When the inflammation and fever are calmed, a derivative action to the skin should be promoted by blisters, sinapisms, &c.: astringents and bitters should be given at the same time. Might not creosote be useful?

In the majority of cases, however, no treatment is of any avail, except perhaps in protracting the morbid action.

Let the physician not be deceived by occasional intermissions of the symptoms: they are greatly fallacious, being quickly followed by all the former distress. When the stage of paralysis has come on, the case is utterly hopeless. —*Heidelberg Klinische Annalen Band. V. hefte* 3.

---

### Surgical Clinique of M. Lisfranc.

### *Fistula Lachrymalis.*

In the ward St. Louis of our hospital (La Pitié), there is a patient who else-

---

* From the prevalence of acid in the secretions of the stomach and bowels, we might expect that alkaline remedies had been indicated in the treatment of this disease. We can confidently state from experience, that when such symptoms as have been described by Dr. *Iselin* to be the accompaniments of *ramollissement* of the stomach are present, minute doses of magnesia, or of chalk or soda, with or without opium according to circumstances, will be found to be the appropriate remedies: a blister on the epigastric region at the same time should not be omitted.—*Rev.*

where has had the operation for this disease performed upon him. The canula, left in the nasal canal, has ceased to be pervious; and, an abscess having subsequently formed at the lower part of the angle of the eye, the pus continues to escape by a small fistulous opening. The question comes to be, will it be necessary to extract the canula? This step may be attended with very great difficulty; the surgeon is sometimes obliged to desist from his attempts. In the majority of cases, it is better not to make the attempt. Ten years ago a patient, who had a canula in his nasal tube, and in whom there was considerable inflammation at the inner canthus, came under my care. I determined to extract the canula; but, previously to doing this, I deemed it right to subdue the inflammation by applying leeches over the mastoid process and poulticing the inflamed part—as it is an excellent axiom in practical surgery always to operate on tissues that are as healthy as possible. When the inflammation was subdued, the canula became permeable, and the patient was cured after the subsidence of the swelling. In the present case, the inflammation has ceased, but a fistula remains. Emollient injections have been ordered to be used; and, in the course of two or three days, we shall pass a stylet along the canula to clear it. If the ulcerated opening does not heal, we shall touch it with the nitrate of silver; but it will most probably cicatrise, as soon as the tears do not pass through it....... Six days afterwards this prognosis was verified, and the patient left the hospital cured.

*Phagedenic Ulcers.*

In the two cases at present under treatment, remember (says M. *Lisfranc*, addressing his pupils) that we have not limited our treatment to merely applying the liquid proto-nitrate of mercury to the surface of the sore. In these, as in all other similar, cases, when the ulcers have an inflammatory appearance, we premise the detraction of blood from the arm; and thus we obviate the increase of irritation and general fever that might follow the application of the caustic.

By adopting this line of treatment, the ulcers in these two cases speedily advanced to cicatrisation; but then, without any discoverable cause, they again relapsed to their former condition. Subsequently however they have re-began to heal; and little now remains for us to do; for both will speedily be well. In one of the cases, the amendment may be dated from the period when he became affected with erysipelas, which was epidemic in the hospital at the time. The inflammatory action seems to have changed the mode of vitality in the tissues. Now the operation of a caustic, applied lightly over the surface of a part only of an unhealthy sore, may be somewhat similar to that induced by erysipelatous inflammation. Our object is not to produce an eschar, but to modify the vitality in the parts around the ulcerated tissues.

*White-Swelling of the Wrist with Fistulæ.*

Amputation of the fore-arm had been recommended to the patient at another hospital. M. *Lisfranc*, however, put him on a course of the muriate of barytes, according to the formula of M. *Pirondi;* and under this treatment the case eventually recovered. From the two fistulous openings over the carpal joint, several small portions of decayed bone escaped; and, in the course of a short time, no traces of any denuded bone could be detected by the probe.

*Engorgement of the Mamma in a Man.*

This is a rare occurrence in men; in the present case, the swelling came on spontaneously, without any previous injury of the part. In treating the case, we have acted in conformity to the distinction which we have so often insisted upon between the condition of sub-inflammation and that of non-inflammation. At first there was a sub-inflammatory action, denoted by slight increase of heat and swelling. On two occasions, 30 leeches were applied around the swelling, and then emollient poultices were used; hemlock pills were taken at the same time internally. Under this treatment, the pains ceased, and the swelling diminished very considerably. Frictions with the ioduret of lead ointment were then employed, and compression by means of disks of agaric and bandages were kept up: the use of the hemlock internally was continued.

"Attend," said M. *Lisfranc,* "to this case, and you will be able to judge of the practice of those surgeons who think of nothing but of excision in all cases of white swelling or engorgements, who call amputation of a mamma a trifling matter, and who cannot understand that the remedial means which *we* employ, even when they do not succeed in preventing the necessity of an operation, have at least the effect of rendering the risk of it much less, by counteracting the unhealthy disposition which unquestionably is apt to lay the foundation for a relapse."

*False Anchyloses.*

You are aware that a year or two ago the practice of violent extension, by means of a machine, in cases of false anchylosis, was highly spoken of by some of our surgeons: we however resisted the almost general favour with which this new mode of treatment was received; and what we predicted of its results has unfortunately proved to be too correct. More than a month before it was utterly abandoned, we had said to its admirers, "you will at least produce a sprain."* You know, if we have been wrong. During that time, we treated, with slow and gradual extension, two patients, in one of whom anchylosis followed a cured white-swelling, and in the other a wound of the joint. You have seen both patients walking about with their limbs extended. Of the other two patients, at present under our care, one came into the hospital for an abscess in the iliac fossa extending down under Poupart's ligament to the upper part of the thigh: this abscess, after being freely opened, healed up favourably; thanks to the application of a number of leeches along the *trajet* of the abscess, after it was opened. But two anchyloses remained: one of the hip and the other of the knee-joint. The second patient, in whom the anchylosis was the result of a cured white-swelling, was submitted to the same mode of treatment. Although the extension, by the machine we used, was slow and very moderate, some degree of inflammation of the joint was induced, which obliged us to discontinue its application for a time, and to have recourse to ordinary means. In a few days the inflammatory symptoms were reduced, and the extending machine was again applied. At the present time, we have succeeded in effecting an almost complete *redressement* in both cases.

*White Swelling.*

We find here the application of the beautiful axiom of Hippocrates: *experientia fallax.* This patient has come to ask our assistance for a white-swelling of the knee-joint, accompanied with much pain and heat. We have had recourse to antiphlogistic remedies, taking care to employ with discretion evacuations of blood, in order that we might not injure the ground on which we had

---

* M. *Lisfranc* is here alluding to a mad proposal of a surgeon in Paris to place an anchylosed limb in a certain machine which he had contrived for the purpose, and by which it might be perfectly extended in the course of one or two sittings! As a matter of course, several cases in which a cure was effected were related! We often wonder how the poor patients in the French hospitals allow themselves to be experimented upon in all sorts of ways. There is M. *Guerin,* at the present time, boasting of having divided upwards of 30 muscles and tendons in the same patient, and at one time, for the cure of various deformities! A gentleman, recently returned from Paris, told us the other day that it is by no means an uncommon thing when cupping is ordered, to see one of the *internes* make several cross incisions with a scalpel, in place of using the cupping scarificator.

to carry on the war: subsequently we have used discutients, when the chronic state of the disease was definitively established. For a time our success seemed complete; the pain and swelling had nearly ceased; when, most unexpectedly, and without any appreciable cause, these symptoms, accompanied with effusion into the joint, returned as severely as ever. The pains yielded for a time to the endermic use of the muriate of morphia; but again they became most distressing. We shall be obliged to amputate the limb; for in all probability there is erosion of the cartilages, and possibly caries of the bones. Nothing is more insidious than the prognosis of chronic swellings of the joints. Here is a second case:

A young man fell upon his knee, four years ago; the slight inflammation which followed was readily dissipated by the use of leeches, &c. The symptoms however returned every now and then; and ultimately the joint became permanently engorged. The lymphatic constitution of the patient forbad the use of very active depletory remedies; at first they produced most satisfactory results; and after a relapse of the symptoms, the employment of the mercurial ointment, according to the plan recommended by M. *Serre d'Ures,* again gave hopes of a decided amendment. This however was only temporary; and we therefore suspected that there must be, in some part of the system, a principle or element which nullified all our exertions. We suspected the existence of tubercles in the lungs; and, *dans une grande consultation,* this suspicion was proved to be, alas! too correct.

*Contraction of the Rectum; Indurations within the Gut, &c.*

We are convinced that, in a far greater number of such cases than is generally supposed, the local disease is of a venereal origin. We must not allow ourselves to be misled by the assertions of the patients that they never had syphilis; for often they will deny it altogether, or so mis-state the facts as to impose on the surgeon.

On the other hand, some surgeons have confounded the venereal disease with genuine cancer of the rectum; and even the great *Dessault* has recorded cases of cure of what he calls cancer. In the present case, our patient says that the only sexual disease he has had for the last four years has been gonorrhœa; nevertheless I gave him mercury internally, and ordered him to have pieces of lint, gradually made larger and larger, introduced up the gut, and kept there at first for a short time, and subsequently for a longer period. Gum-elastic bougies were afterwards substituted for these; and the result has been that now only a few small isolated indurations remain.

In another, but a much more severe and protracted, case of the disease, which has been in the hospital ever since last year, the gut was contracted beyond the reach of the finger; it was also hard, *bosselé,* and adherent to the subjacent tissues, which were then in a state of engorgement; three fistulæ traversed the indurated tissues.

As this patient had been affected with syphilis, we administered mercury at the same time that we used local remedies; and now very slight traces of his disease remain.

I may here mention that, in the year 1829, I admitted a case of indurated and ulcerated rectum, which I considered to be cancerous and to require the excision of the lower part of the gut. As the weather was excessively cold at the time, the operation was deferred, and discutient remedies along with compression were resorted to: the cold weather ceased, but there was then no longer any need of an operation! he was completely cured!! The difficulty of distinguishing cancerous from non-cancerous disease is common alike to the rectum and the uterus.

(*Remark.*—This admission of M. *Lisfranc* will account for the number of operations, often quite unnecessary, which are performed by the French surgeons. A vast number of cases are given over by them to the knife, where a little patience and the use of appropriate constitutional remedies would most assuredly effect a cure. The contrast between French and English surgery in this respect is most favourable to the scientific skill and humanity of our practitioners.—*Rev.*)

*Compound Fracture of the Leg.*

The upper fragment of the tibia projected for at least three inches through the wound. As the bone was not deprived of its periosteum, we reduced it without sawing any portion of it off: there was a vast effusion of blood, and great inflammatory tension in the part. By employing our usual method we brought every thing, with the greatest simplicity, into a quiet state. (Bleeding from the arm on the first day; local bleeding, as a revulsive, for the following four or five days; emollients, and low diet until suppuration commenced.) As the fragments of the bone remained in apposition without the employment of any apparatus, we did not apply one, and merely secured the foot by compresses fixed on each side to the pillow on which the limb reclined. The pus flowed out by the opening, which was on the inner side of the leg. Much embarrassment would have been experienced, if we had not dispensed with the use of an apparatus.

(*Remark.*—We never observe any allusion, in the French writings on surgery, to the simple and admirable plan of treating compound fractures and dislocations by merely laying lint well wetted with the blood of the wound over the seat of the injury, after the bones have been properly reduced. When the blood dries, the lint forms an excellent protection to the wound, completely excluding it from the air, and favouring cicatrisation. The readers of Sir *A. Cooper's* classical work on Fractures and Dislocations need not be reminded of the numerous cases which he has treated successfully in this simple manner.—*Rev.*)

*Immense Enlargement of the Testicle.*

The testicle had increased to at least ten times its normal size. Castration had been recommended to the patient elsewhere. According to our usual practice, we had more faith in a rational treatment. At first antiphlogistic, emollient, and narcotic remedies were used; and then discutients were applied, when all traces of inflammatory action had subsided. Under this mode of treatment, the swelling was reduced nearly four-fifths of its volume. An abscess formed, and was opened; it was of a scrofulous character. Within the last week, another abscess has made its appearance, and we have been obliged to discontinue the use of discutients, and again have recourse to antiphlogistic, &c. remedies.

*Fracture of both Bones of the Leg in an Infant; False Joint.*

Soon after birth this infant had the misfortune to have both bones of the leg broken: the solution of continuity, it seems to us, is too high up to be a mere separation of the epiphyses. However this may be, it has not consolidated; and a false joint has formed. The tendo-Achillis, no longer meeting with any resistance, has drawn the foot upwards and backwards, and keeps it in that position.

Our intention is first to divide the tendon; and then try some means to effect a consolidation.

*Erectile Tumor of the Tongue.*

Our patient was sixty years of age, and, for upwards of thirty, had had on the right side of his tongue an erectile tumor, of the size of a pigeon's egg: it extended from the point of re-union of the anterior with the middle third of the organ to its base. We seized the tongue with a strong hook, and found no little difficulty in preventing its retraction into the mouth: so powerful was the action of its muscles, that we were afraid that the hook would force its way out. The excision was however at length effected, and has succeeded perfectly. For a day or two, there was a tendency to cerebral congestion; but this was relieved by bloodletting.

*Ulcerations of the Cervix Uteri.*

The frequency of this disease is truly astonishing. I have seen at least several thousand cases, the true nature of which must certainly have been mistaken, if the speculum had not been used. Even with the aid of this instrument, ulcerations of the cervix may be overlooked; for, in many instances, there is no appearance on the outer surface of the cervix: it is indeed usually tumefied and puffy; but such a condition is common both before and after menstruation, when no disease exists. By introducing the finger fairly within the cervix, we find that, if any ulcerations be present, its inner surface, instead of being smooth and polished like the pleura, conveys to the finger the same sensation as the mucous membrane of the stomach does, when it is inflamed, villous, and softened.

Another means of diagnosis is by introducing a smooth probe into the cervix and moving it about freely; if any ulcer exists, the surface of the probe, when withdrawn, will probably be spotted with blood.

As to the treatment of ulcerations of the cervix uteri, by far the best method consists in cauterising the diseased surface, after all symptoms of inflammatory action have been relieved, with the acid nitrate of mercury. Some cases are cured by three or four applications of the caustic, while others require as many as eighteen or twenty.

*Remark.*—We need scarcely observe that the practice of using the speculum vaginæ in every woman, who has any symptoms of uterine ailment, is not likely, for very obvious reasons, to be so much in vogue in this country as in France. Fortunately in a vast majority of instances, there is no necessity for it; and as to M. *Lisfranc's* assertion, that he has treated many *thousand* cases of ulcerations of the cervix uteri, we feel assured that, in the greater number of them, there were no ulcers at all.—(*Rev.*)—*Gazette des Hôpitaux.*

---

### Treatment of Fissures of the Anus with Rhatany Injections.

M. *Brettonneau*, the celebrated physician at Tours, seems to have been the first who tried and recommended the use of injections of a strong decoction of Rhatany root in cases of that very troublesome and distressing complaint—fissure of the anus. Constipation is in most cases the cause of the malady, and also the chief impediment to its cure. Now such a state of bowels is very frequently accompanied with a great dilatation of the rectum, especially of that part of it immediately above the sphincter ani. In this dilated portion of the gut the feculent matters are apt to accumulate, and there form an immense hardened mass, the evacuation of which often gives rise to excessive pain, almost as severe as that of child-birth. M. *Bretonneau*, by attending to these circumstances, came to the conclusion that the enfeebled and dilated state of the bowel might be most effectually cured by the use of topical astringents, applied directly to its surface. For this purpose, he used a solution of the extract of rhatany in water, to which some of the tincture of the same was added. Several patients, who had for long suffered both from constipation and from fissure of the anus, were cured of both these complaints by using the Rhatany injection. But it is not only in those cases of fissure of the anus, which are accompanied with a confined state of the bowels, but also in many other cases, that a cure has been effected with the remedy.

M. *Trousseau* writes: " within the last few months, I have treated five cases with this remedy; of these four have been cured. M. *Marjolin* has also succeeded in one case, and M. *Berard* in two. My plan of using it is to order the patient to take an aperient enema every morning, and half an hour afterwards an injection made of a strong decoction of Rhatany root, which he should endeavour to retain; this injection should be repeated in the evening: when the pains attending the act of defecation become abated, one injection in the course of the twenty-four hours will be sufficient."—*Journal des Connaissances, &c.*

---

### SUBCUTANEOUS SECTION OF FORTY-TWO MUSCLES, TENDONS OR LIGAMENTS, IN THE SAME PATIENT.

" I have the honor," says M. *Jules Guerin,* " to communicate to the Academy the account of an operation, which, by its character of generality and its immediate results, appears to me destined to fix in a definitive manner the value of a principle, which I have endeavoured to explain in my memoir on subcutaneous wounds—viz: that wounds made under the skin, and thus out of the reach of the air, are exempt from all tendency to suppurative inflammation.

On the 25th of this month, August, I made, in the case of a young man twenty-two years of age, the subcutaneous division of forty-two muscles, tendons or ligaments, to remedy a series of deformities of the trunk and extremities, induced by the active retraction of these muscles and ligaments. This series of operations required twenty-eight incisions of the skin. The following muscles, tendons and ligaments were divided: the pectoralis major, the brachial biceps on each side, the two pronatores radii teretes, the two radiales antici, the two flexores digitorum sublimes, and the two palmares parvi; also the tendons of the ulnares antici, those of the palmares magni et parvi, and those of the abductores pollicis. Besides these muscles and tendons in the arm and of the elbow and wrist, the following also in both of the lower extremities were divided—at the knees, the sartorius, the biceps, the semi-membranosus, the semi-tendinosus, the rectus internus, the fascia lata and external lateral ligaments, and, at the feet, the tendo-Achillis, the tibialis anticus, the extensor communis, the extensor proprius pollicis, and the peroneus anticus.

*Voici!* the immediate results of these operations:—The patient experienced only *mediocre* pain and fatigue; he uttered no complaint during the performance of the operations, which occupied a full hour. Soon afterwards he fell asleep; and the following night and next day he remained quite tranquil. No sign of inflammatory action any where presented itself; and by the third day the twenty-eight wounds were completely healed. Surely such a case as the present must convince every one of the perfect innocuity of the subcutaneous division of muscles and other parts."—*Gazette Medicale.*

---

### POST-MORTEM EXAMINATION AFTER SUCCESSFUL LIGATURE OF THE COMMON ILIAC ARTERY.

M. *Salomon,* professor of surgery at St. Petersburg, tied the left common iliac artery in a case of aneurysm in the groin: the success of the operation was complete. Ten months afterwards, the patient died from the formation of a large abscess in the iliac muscle of the affected side. The following description is given of the state of the iliac and femoral arteries, observed after an injection had been thrown into the descending aorta.

" The injection had passed into both lower extremities; the left common iliac artery was found to have been tied at about an inch above its division; from this point to its junction with the aorta, it was converted into a firm ligamentous cord. A small quantity of the injection had reached the left external iliac by the hypogastric or internal iliac, which communicated freely with the corresponding vessels on the other side. The left femoral artery began to be injected at about 54 millimetres below Poupart's ligament. The right common iliac, external iliac, and femoral arteries, with their branches, were much dilated; likewise the left lower lumbar arteries, which anastomosed with the circumflex artery of the ileum. The middle artery of the sacrum also, but principally the branches of the ischiatic and common pudic, and also the vesical and hæmorrhoidal vessels on the left side, were found much larger than usual; they formed in the pelvis a considerable vascular network, which communicated freely with the vessels of the opposite side. The anastomoses between the branches of the ischiatic and of the deep femoral artery were most conspicuous on the back of the limb. The left inferior epigastric—which, as well as the circumflex artery of the ileum, was obliterated at its commencement—was contracted, and received its blood in part from the superior epigastric, and in part from its fellow on the right side. The left obturator artery was considerably dilated, and communicated in the thigh with branches from the femoral. The deep femoral and all its branches were filled with injection, although the vessel was ligamentous at its origin.—*Gazette Medicale, Aout.*

---

### Aneurism of the Carotid: Ligature beyond the Tumour: Cure.

A woman, 63 years of age, had a pulsating tumor on the left side of the neck, and immediately opposite to the sterno-clavicular articulation, which very seriously distressed her breathing. This aneurismal tumor was considered to be of about three years' standing. As it was situated so low down the neck that it was impossible to put a ligature around the artery between the seat of the aneurism and the heart, M. *Colson* determined to try the operation of *Brasdor*, that of tying the vessel beyond the tumor. The success of the operation was eventually complete; the pulsations of the swelling became gradually less and less, and the dyspnœa was effectually relieved. It is now a twelvemonth since the operation was performed; the tumor is reduced to the size of a small nut, and still communicates very feeble pulsatory movements.

When this case was communicated to the Royal Academy, M. *Larrey*, who had been appointed to report upon it, expressed his utter disapproval of the Brasdorian operation, in spite of the success which attended the present and a few other cases. He had in his own practice obtained such satisfactory results from the application of ice and of moxas in the treatment of aneurismal tumors, that, in his opinion, no other means except these should be used, when the ordinary operation cannot be performed. He alluded to a case of aneurismal varix between the external iliac artery and vein, which he cured in this way, although the tumor was so large that it reached, and even passed, the level of the antero-superior spine of the os ilii.

M. *Blandin* was unwilling to go so far, as M. *Larrey*, in condemning the Brasdorian operation in all cases without exception. No one indeed can deny that the application of ice and of moxas over aneurismal tumors has proved quite ineffectual in a certain number of cases. Then what is the surgeon to do? Is he to refuse to try the effect of an operation, which is certainly not a good one, but which has succeeded a few times?

M. *Velpeau* agreed with the observations of M. *Blandin*. The Brasdorian operation has now been performed about 20 times, and five or six of the cases have terminated favourably. As far as he knew, it had been performed in France only twice—by M. *Dupuytren* and M. *Langier*—and in both cases unsuccessfully, before the present case of M. *Colson.* The method of M. *Larrey* ought certainly to be fairly tried before we resort to so uncertain an operation as that of tying a large artery beyond an aneurismal sac. Perhaps no artery is so favourably situated for the Brasdorian operation as the common carotid, seeing that no branches are given off between the sac and the point at which the ligature is placed. In two or three cases, this operation has succeeded even in cases of aneurism of the arteria innominata.

---

### Varicose Aneurism between the Internal Carotid Artery and the Internal Jugular Vein.

The following description of the appearances found on dissection, in a case of gun-shot wound of the mouth, which proved fatal about a twelvemonth after the accident, by inducing cerebral disease, will be read with interest:

.. ......... "After carefully dissecting the superficial muscles of the neck, and having removed the *sterno-cleido-mastoideus,* the *hyo-scapularis,* and the three small muscles which are attached to the styloid process, I sawed the inferior maxilla across and disarticulated it from the socket. Having done this, it was easy to follow the carotid artery and the internal jugular vein along their whole course. At the point situated immediately behind the angle of the removed jaw, we perceived the ball lodged within the jugular vein. The parietes of the vessel from this point upwards to the base of the skull were much thickened and had acquired nearly the consistence and firmness of the coats of an artery; the lower part of the vein retained its natural thinness and shining aspect. On tracing the internal carotid artery it was found to form a pouch of the size of a pigeon's egg just before entering the canal in the temporal bone. This pouch contained several coagula, which were partly sanguineous and partly fibrinous. At its base, it was found to communicate with the internal jugular vein; and from this point the artery retained its ordinary form and dimensions. In passing a probe from the aneurismal pouch to one side it readily entered the cavity of the internal carotid; and when it was directed to the left side, it entered the jugular vein just where the ball was lodged, and where it had become so adherent that it could not be pushed either up or down."

Those who may wish to know the particulars of this very curious case will find it recorded in one of the July Numbers of the *Gazette Medicale.*

---

### Ablation of the Clavicle.

In the number of the Gazette Medicale for last July 18th, we find short reports of two cases, where the entire clavicle was removed in consequence of necrosis. In both cases, the bone had become quite moveable; and in one of them it would seem that no incisions were necessary, as the bone was removed with a pair of forceps through an ulcerated wound, that had long existed. In the other case, the necrosed clavicle was cautiously separated from its attachments to the sternum and the first rib, and then from its attachments to the acromion of the scapula: these steps were effected without much difficulty, and the entire clavicle was then easily brought away. Both of these cases occurred in scrofulous children; and the two young patients not only recovered their health perfectly, but also retained an almost complete use of their arms.

---

### Notice of the Medicinische Jahrbucher.

We have received the 29th volume of this Austrian journal—*one of the very best that is published in Germany*—from its chief editor, Professor *Rosas* of the University of Vienna. It consists of four numbers, one appearing every three months. The contents are arranged under several heads.—1. Original essays and communications. 2. Medical statistics. 3. Analytic notices of the most eminent works; and 4. Miscellaneous extracts from the leading medical journals of Germany and of other countries. As usual with all German writings, this Journal is characterised rather by curious reports and elaborate erudition, than by strictly practical information. We miss the clinical observations, the useful hints and precepts, and the well-selected extracts from new books, which to an English reader constitute the value of a medical journal. The following excerpta will explain our meaning, and are a fair specimen of the contents of the "Medicinische Jahrbucher."

### Case of Menstrual Flux in a Man.

A tall but rather delicately formed young man, 21 years of age, and whose health was on the whole perfectly good, observed for the first time in February 1838, that there was a spontaneous discharge of blood from the urethra: this discharge continued for four days. When it ceased, he found himself lighter and altogether better than he had been for some time before. From this period, the discharge, always preceded for two or three days by headache, vertigo, &c. returned every fourth week, remaining each time two or three days. His health was always better immediately after its cessation. Various means had been tried to stop this anomalous evacuation, but without avail. (It is not stated how long the man had been subject to it before Dr. *Julius,* the reporter of the case, saw him.)—*Rev.*

### Examination of the Auditory Organs in a Deaf and Dumb Person.

A youth, who had been deaf and dumb from his infancy, was admitted into the institution for such invalids at Vienna, when he was eight years of age, and he died there in his twelfth year. The dissection of the encephalon, and more especially of every part connected with the auditory function, was performed with great care, and the report is given at full length. The following is a summary of the more remarkable abnormal appearances.

" This examination of the organs of hearing shews that the abnormal condition of these parts depends upon a congenital defective formation of the bony parts, some of these being imperfectly developed, while others are developed in excess. To the *first class* may be referred the absence of the *promontory* and of the *foramen rotundum,* the complete fusion, so to speak, of the *stapes* with this foramen and with the Fallopian canal, the want of the pyramidal-formed elevation, by which is caused in part the deficiency of the *stapedius* muscle, and the imperfect formation of the cochlea. To the *second class,* the greater accumulation of osseous matter in every part of the cranial bones, and the superfluous formation of a roundish little bone between the bony process of the *incus* and the head of the *stapes.* The circumstance too of the want of the usual proportion between the middle and the posterior hollows of the cranium, in consequence of which the whole skull has somewhat of an oblique direction—this has been observed in two other cases of deaf and dumb persons—and, on the contrary, the perfectly normal formation of the nerves, which are expended on the tympanum and on the labyrinth, deserves to be noticed.

From these facts it seems that, in the present case, at least, the deafness was owing not to any defective state of the nervous apparatus of the ear, but only to some irregularities in the formation of the bony parts of the tympanum and of the labyrinth."

### ANÆSTHESIA OF THE TRIGEMINUS NERVE.

A woman, 42 years of age, had the misfortune to fall and strike the back of the head on the edge of a stair. A year afterwards the catamenia ceased altogether, and from this time she began to suffer from frequent attacks of most violent sneezing. No unusual appearance could be detected in the nostrils; and it was therefore suspected that there was an irritation of the fifth pair of nerves in the cranial cavity. Along the course of the first and second divisions of the trigeminus there was no loss of sensibility; but the third division was decidedly *anæsthetic.*

The left half of the under lip, both on its inner and its outer surface, and the left half of the chin, were quite insensible, even when pricked deeply with a needle: the inner portion of the muscle of the corresponding ear and of the meatus auditorius were equally dead to all impressions. The teguments of the left temple near the hair, and also the entire left half of the tongue, were perfectly insensible alike to injury and to changes of temperature: this side of the tongue too had lost its sense of taste. But when the skin of the temple near the fore-head was pricked, the patient immediately complained—in consequence of this part being supplied with twigs from the *frontalis* nerve. On the right side all the corresponding parts were quite sensible; and even in the left eyelids the other sensory nerves retained their integrity, both as respected sensation and power of motion. The organic and nutritive functions of all the parts, which were insensible, were not at all impaired. The patient eventually died of dropsy.

*Dissection.*—At various points on the surface of the brain there was an exsudation of lymph; and on the lower surface of the posterior lobe the cerebral substance was found in a state of *ramollissement,* to the extent of an inch or so. The third, or submaxillary branch of the trigeminus pair on this (the left) side, where it entered the foramen ovale, appeared to be enveloped with a red vascular network, composed partly of fibres and partly of transparent vesicles. On close inspection, it seemed to be either an exsudation on, or an hypertrophied state of, the neurilema: the substance of the nerve itself was swollen, of a yellowish colour, and somewhat harder than it usually is. But it was only that portion of the third branch, which arises from the Gasserian ganglion, that was so altered. The motory portion on the inner side was unchanged, and coalesced with the larger division beyond the diseased point. The various twigs to the pterygoid and buccinator muscles, to the temple, the tongue, and the lower jaw, were throughout in a normal condition, as well as the third branch of the right trigeminus, and also the glosso-pharyngeal on both sides.

### CURIOUS CASE OF ENCYSTED TUMOR ON THE EDGE OF THE LIVER.

A delicate woman, of about 30 years of age, had observed, for some time before her marriage, a soft uniform swelling in the precordial region, which had gradually appeared without any appreciable cause, was always most prominent when the stomach was distended with food, and gave rise to a certain feeling of oppression and uneasiness at that time. During her first pregnancy she found that, immediately after the morning attacks of vomiting, the swelling was always much smaller than it was just before; but when these ceased, that it became larger and larger until it acquired the size of a goose's egg. After delivery, the swelling subsided altogether; but, during her second pregnancy, it re-appeared, and caused greater uneasiness than on the former occasion.

The operation of an emetic always caused a diminution in its size, but when this was over, the swelling gradually returned. The woman became phthisical and died.

*Dissection.*—The lungs were loaded with tubercles in various stages of development. On opening the abdomen, the upper part of the left lobe of the liver was found to be adherent to the cardia by a tumor of the size of a man's fist. This tumor proved to be of an hydatidic character; and its parietes consisted of three distinct membranes, which inclosed a quantity of yellowish serum: it was an acephalocyst of *Laennec.* At one point of its circumference there was a fistulous opening, which communicated with the cavity of the stomach, and by which therefore its contents were evacuated during the efforts of vomiting.

### Nux Vomica in the vomiting of Pregnant Women.

Dr. *Kroyher,* of Presburg, assures us that minute doses of the nux vomica, given in some aromatic or in cherry-laurel water, are a *specific* remedy against the troublesome vomiting, to which many women are subject during the early months of pregnancy. In order to insure success, the bowels must be kept in a gently open condition, but neither purged nor constipated. The author says, that the effects of this remedy are certain, provided the vomiting is the result of pregnancy alone, and is not dependent on any morbid state either of the stomach or of any other organ. The dose recommended is from two to four drops of the tincture—the strength of this is not stated—to be gradually increased to ten, twelve, or eighteen drops every morning in bed, and again in the evening. In many cases it proves quite successful within a week or even a shorter time; in other cases its use must be continued longer.

### Case of Spontaneous Hydrophobia.

A middle-aged man, while under treatment for venereal complaints, was unexpectedly seized with a complete inability to swallow any thing either solid or fluid, and with such well-marked hydrophobia that the mere sight of fluids brought on strong convulsions. His countenance betrayed the most intense anxiety, and every now and then he gasped at the air whenever the gentlest breath of it was blown upon him.

Neither he nor any of his acquaintances remembered that he had ever been bitten by a dog or any other animal. As he could not swallow powders of calomel and belladonna, the latter substance was applied to a blistered surface on the epigastric region, and mercurial ointment mixed with extract of belladonna was rubbed in along the spine. The poor fellow made the most violent efforts to swallow food and medicine; but all without avail: he was immediately seized with the most excruciating convulsions, during one of which he sprung out of bed like a maniac, and with foaming mouth tried to bite the attendants; while standing, he fell down and expired. The hydrophobic symptoms had lasted for about 60 hours. Permission could not be obtained to examine the body.

The preceding case is the more remarkable, as the spontaneous hydrophobia or dread of fluids was not merely a symptom, but the actual and essential disease, and terminated with genuine rabies. It is therefore beyond all doubt the *rabies canina* may be developed as a primary disease in the human being. The circumstance of priapism having preceded the hydrophobia appears to confirm the causal connexion, although this is disputed by many, between the disease and excitation of the generative organs. (This case is taken from *Caspar's* Wochenscrift, No. 24, for 1839.)

### ON THE EXTERNAL AND INTERNAL USE OF VERATRINE.

The principal action of this very potent substance seems to be on the spinal-marrow; for, soon after it has been swallowed, the person begins to experience a dull burning pain in the sacral region, various uneasy feelings through the abdomen, increased watery and slimy evacuations from the bowels, but rarely any diuresis. If its use be still continued, it causes dryness and a sense of burning in the mouth, intense thirst, nausea, vomiting, bloody stools, coldness of the limbs, trembling, syncope, delirium, and paralysis: the urine is usually scanty, thick, and of a deep red colour. The surest antidote to these symptoms is strong coffee with lemon-juice.

The veratrine, endermically used by sprinkling half a grain on the epigastric region deprived of its epidermis, excites nausea, sense of tightness in the chest, electric-like dartings through the chest and abdomen, and painful twitchings in the limbs. In some cases of palsy it has been used with decided advantage; but, as it is certainly inferior to strychnine, we can generally dispense with it. The best mode however of using the veratrine is friction with an ointment containing from ten to twenty grains, rubbed up with an ounce of lard: its peculiar electric-like local effects are most easily obtained in this way. It has been used with excellent effects in all forms of purely-neuralgic suffering, especially in *hemicrania, ischias nervosa, neuralgia facialis, asthma, convulsions,* and in some most excruciating neuralgiæ arising fron calculus in the kidneys:—in many cases, combined with the extract of belladonna, it is still more efficient.

Another effect of veratrine is the excitation and regulation of anomalous actions (thätigkeitsausserungen) of the nerves proceeding from the spinal marrow. If an ointment—composed of from two to four grains and an ounce of lard—be rubbed along the spine, twice a day for five, six, or eight weeks, much relief will often be experienced in weakness of the lower limbs, (especially if this has been the effect of seminal irregularities,) in rheumatic pains, in debility of the bladder and sphincters, in menstrual cramps,* in convulsive affections of the urinary organs, in suppressed hæmorrhoids, in pertussis, and probably also in diabetes mellitus. Dr. *Reiche,* the writer of these remarks, has never observed that the veratrine has any diuretic effects; although he has certainly found that many of the distressing symptoms of hydrothorax and hydrops-pericardii are much relieved by its external use. That it exerts emmenagogue powers cannot be disputed. It seems also to stimulate the absorbent vessels of the part, and it has therefore been used with advantage in the dispersion of some subcutaneous tumors.

In a subsequent article, taken from the *Medicinishe Zeitung,* there is reported a case of severe intermittent neuralgia of the frontal nerves, which, after resisting the internal use of quinine and other remedies, yielded at once to the application of veratrine ointment—two grains to the drachm of lard—to the forehead previously denuded by a blister of its epidermis. The effect was most rapid and well-marked.

---

* Some English practitioners have of late recommended frictions with veratria ointment over the sacrum as a sovereign remedy in certain intractable cases of dysmenorrhœa. We have no experience of it ourselves; but we are in the daily habit of using the belladonna plaster in such cases with admirable effect.—(*Rev.*)

### Case of Obliteration of the Aorta.

A soldier, who had served in all the German campaigns from 1790 to 1815, had for five years before his death suffered a great deal from difficulty of breathing and cramps in the stomach :—these latter were often excessively severe, and brought on such violent vomitings, that all food was rejected. To these symptoms were gradually added most distressing palpitations of the heart and anasarcous swellings of the lower limbs. Under the use of the *magisterium bismuthi* and digitalis, the patient experienced great relief: the pulse however continued to give out a peculiar *whirring* sensation, which lasted to the period of the patient's death; this occurred very suddenly, while the patient was sitting at table.

*Dissection.*—The brain was very soft; its vessels empty of blood; and the basilar artery was ossified : four ounces of serum were found at the base of the cranium.

The heart was greatly enlarged and hypertrophied : the valves however, in both cavities, were healthy. The aorta from its commencement to the giving off of the arteria innominata was much dilated, and the latter vessel was nearly twice as large as usual. The left subclavian artery was in a similar condition, but the left carotid was unaffected. The two coronary arteries of the heart were completely ossified to the extent of three inches or upwards.

From the point of origin of the arteria innominata, the aorta was considerably narrowed and became more and more so : where the ductus Botalli joins the arch, it was not above half an inch wide; and, just beyond this point, its canal was completely obliterated for half an inch in extent, by the cohesion of its parietes. The thoracic and the abdominal aorta was scarcely so large as it is usually in a child of 10 years of age.

The intercostal arteries, which were the first branches that were given off below the point of obliteration, were enlarged to the fourth of an inch in diameter, and inosculated freely with branches of the internal mammaries. It was chiefly through these arteries that the interrupted circulation of the blood had been again restored. The pulmonary arteries seemed to be somewhat dilated, although the lungs themselves were in a perfectly healthy condition.*

The case, which we have now briefly reported, has considerable resemblance to that recorded by Dr. *Graham* in the 5th volume of the Medico-Chirurgical Transactions, and which occurred in a youth 14 years of age, who died of the effects of pneumonia. On dissection, the heart was found to be greatly enlarged and the walls of the left ventricle excessively thickened; all the valves however were healthy. At its commencement, the aorta was considerably dilated; but below the giving off of the left carotid and the subclavian arteries it was much contracted : the contraction extended as far as the point where the *ductus Botalli* joined the aorta, where it was found to be completely obstructed for two or three lines in extent. The parietes of the aorta were neither thickened, nor in any degree altered. The circulation of the blood had been maintained by the enlarged anastomoses between the upper intercostal and the scapular and internal mammary arteries on the one hand, and the lower intercostal and the epigastric on the other. Beyond the obliterated point, the aorta resumed its normal size and diameter.

Other cases of obstruction of the aorta are on record. *Stenzel*, in his essay De Steatomatibus in principiis Aortæ Repertis, mentions the case of a man,

---

* There is a very good engraving attached to the report of the case, so that the reader may judge exactly of the situation of the obstruction and the relative changes in the size of the different arterial branches.

who, although subject to dyspnœa, asthma, and palpitations of the heart, was robust and vigorous. Yet, on dissecting the body, two large tumors were found in the arch of the aorta, the tube of which was completely obstructed in consequence. Another case is related in the second volume of *Dessault's* Journal of Surgery: in it however the obstruction, which was situated immediately beyond the arch, was not complete, although so great that a writing-quill could not be passed through it. Sir *A. Cooper* too has recorded a somewhat similar example, which occurred in a man, 57 years of age, who had for many years been subject to a cough, dyspnœa, &c. A third analogous case will be found in Professor *Otto's* Neue Seltene Beobachtungen.

---

### A Poem on Syphilis.

One of the novelties of French literature, during the last year, is a poem in two cantos on the *popular* subject of syphilis, by M. Barthelemy of Paris! "In the present day, when all other themes," says he, "are exhausted, none seemed altogether more virginal than the one which I have selected." The work is addressed both to savans and the people of the world. How far the literary public will approve of our author's choice remains to be seen—some indeed will be inclined to cry out on the mere inspection of the title-page, and say with *Boileau,* that "le lecteur Français veut etre respecté." It is however surprising how M. *Barthelemy* has discoursed so learnedly upon the mysteries of his subject, without offending either decency or good taste: at least so says one of his compatriot reviewers—"the most strict father of a family may put this work into the hands of his son without any alarms of conscience; nay, he may even encourage him to meditate on the pictures which it presents, pictures which are at once hideous and salutary."

The poem commences with a history of the disease: the author does not pretend to decide whether it was known to the ancients, or whether it came from the new world,

———"Vengeant sur nous sa liberté mourante,
L'Amerique ait conquis l'Europe conquerante."

Whatever be its origin, it is but too true that, now, not a corner of the world is exempt from the scourge:

"Invisible et present, comme l'air qu'on respire,
Ce grand empoisonneur tient tout sous son Empire.
Nulle digne qui puisse arreter ce torrent;
Il saisit, á la fois, le docte et l'ignorant,
Le riche en son hotel, le pauvre en sa cabane,
L'empie et l'homme saint qu'abrite la soutane,
Le vieillard, l'enfant meme, atteint souvent d'un mal
Dont il n'est pas lavé par le flot baptismal;
Et peut-etre aujourdhui, parmi l'espece humaine,
Il n'est pas un seul homme, et dans l'homme une veine
Où, quoique bien souvent encore non revelé,
Le virus destructeur ne soit inoculé." &c.

The degeneration of the present race of mankind in their thewes and sinews from their forefathers, whose armour we pigmies of the nineteenth century can scarcely lift, not to mention the numberless abortive-looking mortals we daily see, with narrow chests, pale faces, hollow eyes and deformed limbs, "qu 'attend orthopædie," are all ascribed by our poetical author,

"Au germe de mort infiltré dans leur sang."

He seems to have a high opinion of the antidotal power of certain remedies; for in the second canto, which is entitled *Le Remede,* the first being entitled *Le Mal,* he tells his readers that the scientific physician can confidently predict the date of cure to his patient:

Et ce jour le malade, affranchi de souillure,
Se leve et prend son lit, comme dans l'ecriture;
Miracles du savoir, si soudains et si beaux,
Qu'il semble dire aux morts: sortez de vos tombeaux!

He is a decided anti-mercurialist, and trusts exclusively to vegetable remedies. Appended to the work are numerous scientific and well written notes from the pen of M. *Girandeau de St. Gervais.—L'Encyclographie.*

---

### Case of Spontaneous Combustion.

Dr. *Lievin,* one of the surgeons of the French Army at Algiers, was summoned to visit a Moor that had become suddenly ill. He found his patient, a man between 40 and 50 years of age, in a state of profound coma: he was large, very fat, and bore all the traces of an habitual drunkard. He had been missed by his friends for several days, and was at length found lying in the streets in a state of intoxication. He was immediately bled from the arm and leeched very copiously, &c. Two days afterwards he was again bled; and on the fifth day he had so far recovered as to be able to go to the Mosque to return prayers for his convalescence: he returned drunk. Next day he again went out and did not return for three days. This life of inebriety had continued for a month, when Dr. *Lievin* was again called to his house. A horrible spectacle awaited him there: on the ground lay a corpse three-fourths consumed, black, carbonised and exhaling a most offensive empyreumatic smell. The limbs and a great portion of the trunk were consumed. The account which the attendants gave was, that he had been brought home, on the preceding night, drunk as usual, and was put to bed. A smell of burning being perceived in the house some time afterwards, they entered his room and found him suffering from excruciating pains; he said that he was burning all over; he drank freely of water, but found no relief. A blueish coloured flame was observed playing around his body, which exhibited in different parts some frightful wounds. The attendants left the room in horror, believing that he was a victim to some demon, in consequence of his having disobeyed the commandments of the holy Prophet.

The combustion in this case took place by the simple force of the organisation; no body in a state of ignition had been near the patient.—*Journal des Connaissances Medicales. Mai.*

---

### Of the Perforating Ulcer of the Stomach.

The following observations are from the pen of Professor *Rokitanski* of Vienna, where the disease must be more than usually frequent, as our author states that he has seen upwards of 100 cases.

The perforation in the stomach is in general circular and of three lines or more in diameter; the edges of the aperture are sharp and well defined, giving the appearance as if a round piece had been cut out with an instrument. As the loss of substance is always somewhat greater on the inner than on the outer surface, the edges on the former are necessarily attenuated more and more, as they approach the aperture.

The ulcer is almost always found in the pyloric half of the stomach; in one case only the author observed it in the fundus or small cul-de-sac. Most frequently the ulcer is situated about the middle of the pyloric half, generally on its posterior wall, and always near, and often on, the small curvature. The cicatrices of former ulcers are usually observed on the inner surface of the stomach; rarely on the outer. The situation of the ulcer on this part of the organ is more remarkable, as, according to the researches of M. *Rokitanski*, Gastromalacia, or softening of the stomach, is always observed in its cardiac half.*

Ulcers are more rare very near to the pylorus; and the author has never seen them in any part of the intestines, except in the upper part of the duodenum; this however is of very unfrequent occurrence.

Of 79 cases, in 20 the ulcer was situated on the posterior wall of the stomach, in 15 on the small curvature, in 5 on the anterior wall, in 16 at a short distance from the pylorus, in 6 in the duodenum, and in 16 in different parts, as in the anterior and posterior walls, at the same time.†

The size of these ulcers varies from that of a *sou* to that of a five-franc piece, and sometimes it is even larger. Their shape is usually circular; when large, they sometimes take an elliptical form; more rarely still they are irregular. The author is of opinion that the ulcer commences with a circumscribed softening, as an eschar, of the affected part; but the exciting cause of this local mortification he cannot explain. Let it not be supposed that the disease is inevitably progressive, until it proves fatal. The destruction of the mucous membrane, with which the diseasc begins, may be repaired by cicatrisation: the corrosion stops in the submucous cellular tissue, which then becomes of a fibrous character, and unites firmly with the edges of the mucous membrane on the one hand, and with the muscular tissue on the other.

That the cicatrix-looking marks, which we not unfrequently observe on the inner surface of the stomach, are in truth the results of the healing of previously-existing ulcers may be fairly inferred, not only from their shape and their situation being the same, but also from the circumstance, that they are occasionally met with co-existent and side by side—not to mention the fact that those persons, in whom on dissection the cicatrices have been discovered, have always suffered, at some former period of life, from those very symptoms which are known to accompany the existence of an ulcer in the stomach.

When the ulcer does not cicatrise, it extends deeper and deeper, until at length it reaches the peritoneal coat of the stomach, and perhaps eats through this obstruction. If no adhesion has taken place at this point between the stomach and one of the adjacent viscera, the contents of the former escape into the cavity of the abdomen, and inevitably induce a fatal peritonitis. Occasionally indeed the ulceration extends to the substance of the adherent viscus: in this way the author has seen the diaphragm in one case perforated, and in another the base of the lung corroded. A hæmorrhage may thus be unexpectedly induced, which may suddenly occasion the death of the patient.

---

* An account of this disease will be found in a preceding article, taken from one of the German Journals, in a former part of the present Number.

† In 12 cases there were two, in 4 there were three, and in one there were as many as five ulcers at the same time. When there is a plurality of ulcers, they are usually situated, one above the other, on the posterior wall or at the small curvature of the stomach: it is rare to find one at the anterior, and another at the posterior wall—this was observed four times only out of seventeen cases. In one case there were two ulcers in the duodenum, the one situated immediately opposite to the other; and in another case there was one ulcer in the duodenum and another in the stomach close to the pylorus.

With respect to the *symptoms* of ulcer of the stomach, Dr. *Rokitanski* describes three stages of the disease. The *first* is characterised by some form or another of that most Protean malady, dyspepsia, with which the patient may have been afflicted for several years. Increase of gastric pain and distress after food, accompanied with more or less frequent vomiting, is the leading symptom of the *second* stage; while the *third* is characterised by the sudden accession of peritonitic symptoms, induced by the escape of the contents of the stomach into the abdominal cavity. Such an attack may excite the suspicion of poisoning having taken place. *Cruveilhier* has said that the perforation may suddenly take place during a bodily exertion : this may be so. When the opening is plugged up by some adjacent viscus, we cannot, as a matter of course, determine the moment it occurs ; but we may suspect it by the invasion of distressing cardialgia, lasting perhaps unabated for several days and accompanied with swooning, vomiting of blood, &c. The hæmorrhage, as we have already stated, may prove fatal at the time ; in other cases it returns on several occasions. The disease may therefore be said to terminate in one of two ways—either by peritonitis or by hæmorrhage. It rarely becomes chronic, and exhausts the patient by mere debility : occasionally indeed before death dysentery supervenes.

In spite however of the very unfavourable prognosis in every case of ulcer of the stomach, it is most certain that a cure not unfrequently takes place—attested, as we have already stated, by the discovery of circular cicatrices on the internal surface of the stomach in persons who had previously suffered from all the symptoms of the first and second stages of the disease. The disposition to relapses in such cases is very remarkable: this is proved by the presence of one or of several of these cicatrices situated near to, or at, the side of the ulcer. Even when the cicatrisation is complete, the patient usually continues for a greater or shorter length of time subject to dyspeptic sufferings.

As to the *etiology* of the disease, the author says that he has seen it accompanied with irregular hæmorrhoids, with dysmenorrhœa, gout, &c. ;—but he has never observed any distinct *rapport* between these maladies. He is, however, of opinion, that all those diseases which induce repeated irritation of the gastric mucous membrane, and at length occasion an hypertrophe and an increase of the secretion from this membrane, not unfrequently terminate by the formation of an ulcer. Intermittent fevers, when they are accompanied with stomach complaints, have a great influence on the production of this disease. With repect to the sex and age at which the disease is most common, we may state that, out of 79 cases, 46 occurred in females, and 33 in males.

Amongst the *former*, 18 were above 50 years of age, and 15 were under 30; of these last there were three whose respective ages were 16, 17, and 19. Among the *latter*, 12 were above 50 years, and six were under 30.

The phenomena, which we have described as usually present in the perforating ulcer of the stomach, are observed in cancerous disease of this organ also; and hence it is not unfrequent that physicians confound the two maladies together. What adds to the difficulty of the diagnosis is, that they are sometimes associated together in one case. This is the more distressing, as it scarcely leaves a hope for expectation of good from any system of treatment ; whereas, in the uncomplicated form of the perforating ulcer, a cure is not unfrequently effected in its early stage by appropriate and perseveringly used remedies.

Our author mentions, as among the best discriminating symptoms of these two kinds of gastric disease, the absence in the latter of those phenomena which indicate a thickening of the pyloric parietes and a contraction of its canal—such as vomiting two or three hours after taking food, the dilatation of the stomach and its cul-de-sac, and, lastly, the presence of a fixed resisting tumor in the region of the pylorus. The vomiting of chocolate-looking matters is more common in the cancerous than in the perforating ulcer of the stomach: in the former disease too, we not unfrequently perceive among the rejected contents of the stomach portions of the cancerous tumor; whereas, in the latter, the vomited matters are mixed with blackish-brown flocculi; hæmorrhage, also, and occasional intermissions of suffering are certainly more frequently observed in this than in the other case; and, moreover, the perforating ulcer is often met with in young persons, whereas cancer is a disease usually of more advanced life.

Lastly, the absence of all the marks of a cancerous cachexia are to be taken into account in forming our diagnosis.

As to the treatment of the perforating ulcer of the stomach, Dr. *Rokitanski* very properly says, that more may be done by an appropriate diet than by any particular course of medicine. Milk is, on the whole, the best food; a small quantity should be taken every three or four hours. Magnesia, prepared chalk, lime water, &c. are often useful at times, and may be added to the milk.

When this does not suit the stomach, we should try weak broths, mucilaginous decoctions, panadas, and such like articles. The occasional application of leeches, and of some epispastic, such as the strong antimonial ointment, on the epigastric region is useful: also tisanes of chamomile, mint; and, if hæmorrhage should ensue, acids, alum, acetate of lead, kino, rhatany, &c. We should never have much confidence in any amendment, which occurs suddenly; in all probability it will be of but short duration.—*Oesterr. Med. Jahrbucher,* 1839.

---

### REDUCTION OF AN ALLEGED DISLOCATION OF THE SECOND CERVICAL VERTEBRA.

M. *Guerin,* the celebrated orthopædic surgeon of Paris, has recently published the following case in the *Gazette Medicale.*

A young girl fell on the pavement and struck her chin severely: next day she felt considerable pain in the neck, and the head was observed to be inclined to the left side, while the face was turned to the right. This defoimity became gradually greater for several successive days.

Various attempts were made at the time to rectify the displacement, but without avail. Five months afterwards, she was examined dy MM. *Sanson, Marjolin,* and *Bouvier,* the first of whom was of opinion, that there might be an incomplete luxation of the superior cervical vertebræ, while the two latter surgeons declared most distinctly that the second cervical vertebra was luxated, and that all attempts at reduction must be abandoned.

Five weeks subsequently M. *Guerin* saw the patient, and gave it as his opinion that a gradual reduction of the luxation might be tried without danger, and with some prospect of success.

The following was the state of the case at this period: the head was inclined to the left side, and rotated to the right; the spinal column was inclined in a direction contrary to that of the head: there was a projection of the transverse process of the axis at the right side of the neck, above and behind, with a well-marked depression at the opposite side.

According to M. *Guerin*'s views of the case, the dislocation of the second cervical vertebra had probably followed elongation of the ligaments and laceration of the articular surfaces, being ultimately effected by spasmodic contraction of the muscles of the neck. It seemed therefore rational, he thought, to conclude that the vertebra might be restored to its natural position by placing the head and vertebral column in such conditions as would allow the opposite muscles to act with effect, and gradually overcome the forces which had produced and still kept up the displacement. To diminish the spasmodic contraction of the muscles, frictions with tartar-emetic ointment, and extension along with percussion and kneading of the muscles, were assiduously employed.

After a few days, the inclination of the head was diminished by three-fourths, although the deviation of the transverse process remained the same. To rectify the dislocation of the vertebra was now to be attempted, and the following was the plan which M. *Guerin* adopted :—

The shoulders being immovably fixed in the horizontal position, extension was employed with both hands to the middle and most prominent part of the neck; the parts being drawn horizontally, and from right to left, while an assistant endeavoured to rotate the head from right to left at the same time. The effect of these exertions was visibly to diminish the projection of the transverse process of the axis.

The same attempts were therefore repeated thrice a day; and during the intervals, the patient was placed on the mechanical bed which M. *Guerin* employs in the treatment of wry-neck. At the expiration of eight days, the axis could be restored completely to its normal position: but it did not retain it; for, as soon as the muscles were allowed to act, they drew the transverse process backwards, but in a less marked degree than before.

This circumstance, which depended on fracture of the left articular process, and on considerable elongation of the ligaments and articular capsule, gave an excellent idea of the mechanism, by which the dislocation had been produced in the first instance. To remedy the tendency to recurrence of the dislocation, the bandage, used by M. *Guerin* in cases of wry-neck, was applied; and in three months the patient was pronounced to be perfectly cured.

(*Remark*).—We are by no means satisfied that there was so serious an accident as luxation, even incomplete, of the second cervical vertebra in this case. We are indebted for the report to a Number of the recently started "Provincial Medical and Surgical Journal," edited by Drs. *Green* and *Streeter*, to which we wish all possible success.—(*Rev*).

---

### New Method for the radical Cure of Herniæ.

M. *Velpeau*, the ever active surgeon of La Charité Hospital, alludes to his new proposal in the following terms. :—

.......... Among the methods employed in former times for the cure of herniæ, there is one, that of scarifications, which has appeared to me, when somewhat modified, capable of effecting the end with safety and success. The old plan consisted in opening the sac and scarifying it in several places. Modern surgeons have rejected it as both dangerous and useless. Dangerous I admit it to be; but I cannot go so far as to say that it is useless. It is well known that if the peritoneum is scarified, there is induced a secretion of plastic lymph which will determine the adherence of the parietes of the canal. Since M. *Guerin* has shewn that subcutaneous incisions may be made in a great variety of structures without the danger of inducing any suppurative action or any serious inflammation, provided the external air be not admitted into the *trajet* of the wound, it occurred to me that the principle might be extended to the radical cure of reducible herniæ. In one case you have seen me put my proposed plan in practice. After separating and keeping to one side the spermatic cord and vessels, I slipped a small knife under the skin and endeavoured to insert it as far up as the internal orifice of the inguinal canal. My object was to penetrate into the peritoneum, in order to close the herniary sac at its *commencement.* Having pushed the instrument to a sufficient depth, I then moved it about so as to scarify the inner surface of the sac in all directions. The wound in the skin was scarcely a line in extent; not more than two or three drops of blood escaped; and the patient did not complain of any pain. No unpleasant consequences followed the operation; but we are not informed as to the eventual results of it.—*L'Esculape.*

*Remark.*—The plan of subcutaneous incisions in various surgical maladies, as recommended by M. *Guerin,* is certainly well deserving of the attention of surgeons. There seems to be no danger accompanying the operation, even when important tissues, such as the capsular ligaments of joints, are divided—provided the external wound be very small, and the air be prevented from entering. Already we observe that some English surgeons have adopted the practice of dividing the contracted muscles in certain cases of spinal deformity, and apparently with decided success.—(*Rev.*)

---

### On Hospital Gangrene in Paris.

The following letter was recently addressed to the editor of the Bulletin de Therapeutique by M. *Devergie,* one of the medical officers of St. Louis hospital.

Hospital gangrene has attacked not only some of the patients of M. *Jobert,* but also two of the cases in my wards. In one, that of a scrofulous patient affected with swelling of the inguinal glands and numerous abscesses, the application of cinchona and charcoal powder, equal parts, moistened with lemon-juice, succeeded in stopping the disease. The other case was much more severe. There was a leprous ulceration of the upper half of the left cheek, and lower eyelid, extending to part of the forehead and of the right cheek also. The application of the *Canquoin* caustic had the effect of destroying the indurations of the right cheek; but neither this, nor the iodine ointment, the chloruret of sodium, nor various other remedies, did any good to the disease on the left side. In a short time, the wounds inflamed, their surfaces assumed a yellowish-grey hue, the suppuration became copious, and ulcerous pustules formed on the neck and behind the ear. The ulcerations on the face gradually coalesced, until the entire cheek formed one large wound. In vain I applied *Dupuytren's* favorite remedy, the bark and charcoal powder mixed with lemon juice; but fortunately under the use of the acid nitrate of mercury,* diluted with an equal weight of water, the mischief was arrested; in six days, the lupus was destroyed, and the wounds cicatrised with surprising rapidity, so that a perfect cure was effected, with the exception of an ectropion of the left eyelid remaining.

M. *Devergie* is of opinion that the cause of the hospital gangrene in the St. Louis hospital is its exposure to the unhealthy emanations from Montfauçon, (where there is a large *abattoir,* or slaughtering-house, and where there is often collected an immense accumulation of most offensive animal refuse), as the patients in those wards, which were on the other side of the building, seemed to be quite exempt from it.

---

* We presume that this is a solution of the nitrate in nitric acid.—*(Rev).*

